# Guiding seed movement: environmental heterogeneity drives genetic differentiation in *Plathymenia reticulata*, providing insights for restoration

**DOI:** 10.1093/aobpla/plae032

**Published:** 2024-05-29

**Authors:** Taise Almeida Conceição, Alesandro Souza Santos, Ane Karoline Campos Fernandes, Gabriela Nascimento Meireles, Fernanda Ancelmo de Oliveira, Rafael Marani Barbosa, Fernanda Amato Gaiotto

**Affiliations:** Escola Superior de Agricultura Luiz de Queiroz, Universidade de São Paulo, USP, Piracicaba, São Paulo 13418-900, Brazil; Laboratório de Marcadores Moleculares, Centro de Biotecnologia e Genética, Universidade Estadual de Santa Cruz, Rodovia Ilhéus-Itabuna, km 16, Ilhéus, Bahia 45662-900, Brazil; Laboratório de Ecologia Aplicada à Conservação, Programa de Pós-Graduação em Ecologia e Conservação da Biodiversidade, Universidade Estadual de Santa Cruz, Rodovia Ilhéus-Itabuna, km 16, Ilhéus, Bahia 45662-900, Brazil; Laboratório de Marcadores Moleculares, Centro de Biotecnologia e Genética, Universidade Estadual de Santa Cruz, Rodovia Ilhéus-Itabuna, km 16, Ilhéus, Bahia 45662-900, Brazil; Laboratório de Marcadores Moleculares, Centro de Biotecnologia e Genética, Universidade Estadual de Santa Cruz, Rodovia Ilhéus-Itabuna, km 16, Ilhéus, Bahia 45662-900, Brazil; Centro de Biologia Molecular e Engenharia Genética (CBMEG), Departamento de Biologia Vegetal, Instituto de Biologia, Universidade Estadual de Campinas, UNICAMP, Campinas, São Paulo 13083-875, Brazil; Departamento de Ciências Agrárias e Ambientais, Universidade Estadual de Santa Cruz, Rodovia Ilhéus-Itabuna, km 16, Ilhéus, Bahia 45662-900, Brazil; Escola Superior de Agricultura Luiz de Queiroz, Universidade de São Paulo, USP, Piracicaba, São Paulo 13418-900, Brazil; Laboratório de Ecologia Aplicada à Conservação, Programa de Pós-Graduação em Ecologia e Conservação da Biodiversidade, Universidade Estadual de Santa Cruz, Rodovia Ilhéus-Itabuna, km 16, Ilhéus, Bahia 45662-900, Brazil

**Keywords:** Atlantic forest, conservation genetics, Fabaceae, restoration, seed sources, tropical savanna

## Abstract

Forest and landscape restoration is one of the main strategies for overcoming the environmental crisis. This activity is particularly relevant for biodiversity-rich areas threatened by deforestation, such as tropical forests. Efficient long-term restoration requires understanding the composition and genetic structure of native populations, as well as the factors that influence these genetic components. This is because these populations serve as the seed sources and, therefore, the gene reservoirs for areas under restoration. In the present study, we investigated the influence of environmental, climatic and spatial distance factors on the genetic patterns of *Plathymenia reticulata*, aiming to support seed translocation strategies for restoration areas. We collected plant samples from nine populations of *P. reticulata* in the state of Bahia, Brazil, located in areas of Atlantic Forest and Savanna, across four climatic types, and genotyped them using nine nuclear and three chloroplast microsatellite markers. The populations of *P. reticulata* evaluated generally showed low to moderate genotypic variability and low haplotypic diversity. The populations within the Savanna phytophysiognomy showed values above average for six of the eight evaluated genetic diversity parameters. Using this classification based on phytophysiognomy demonstrated a high predictive power for genetic differentiation in *P. reticulata*. Furthermore, the interplay of climate, soil and geographic distance influenced the spread of alleles across the landscape. Based on our findings, we propose seed translocation, taking into account the biome, with restricted use of seed sources acquired or collected from the same environment as the areas to be restored (Savanna or Atlantic Forest).

## Teaser

In this manuscript, we consider that efforts to restore degraded environments may prove ineffective if they overlook the fundamental aspect of Biodiversity: Genetic Diversity. Therefore, to offer insights into Ecosystem Restoration, we investigated a tropical species, *Plathymenia reticulata* , a species that helps restore two important global biodiversity hotspots, the Cerrado and the Atlantic Forest. We report the level of Genetic Diversity in the species, demonstrate how this diversity is distributed and test predictors to elucidate the strong genetic structure found among the remaining populations of *P. reticulata* . Additionally, we offer recommendations for the conservation of the *Plathymenia* genus and its remaining populations. It is crucial to recognize that restoration strategies cannot exist in isolation; they must incorporate genetic approaches to facilitate the reconstruction of robust and resilient communities.

## Introduction

We human beings are responsible for a significant contemporary paradox; although we depend on ecologically balanced ecosystems to obtain goods and services essential to our survival, our activities are the main causes driving the degradation and loss of ecosystems on the planet (Isbell *et al.* 2017; Mitchard 2018). Among the actions that negatively impact ecosystems, leading to the loss of biodiversity and its associated ecosystem services, deforestation and forest fragmentation are particularly notable (Faria *et al.* 2023). In particular, the intense and widespread deforestation has resulted in the loss of at least half of the world’s tropical forests, posing a significant conservation concern for this century (Hansen *et al.* 2013; Edwards *et al.* 2019; Aronson *et al.* 2020). For example, the Brazilian Atlantic Forest, known for its high biodiversity, has currently lost around 28 % of its original extent, with substantial losses in biomass and richness, including tree species (Lima *et al.* 2020; 2024; SOS Mata Atlântica and INPE 2022). Similarly, tropical savannas have faced significant pressures leading to changes and loss of the original vegetation cover due to agricultural expansion and unnatural forest fires (Araújo *et al.* 2012; Colli *et al.* 2020; Santos *et al.* 2021).

Addressing this challenge effectively is one of the most significant contemporary issues, requiring nature-based solutions, such as ecological restoration (Griscom *et al.* 2017; Betts *et al.* 2019). However, for ambitious and urgent global restoration objectives to be achieved, numerous challenges still need to be overcome, for example, obtaining native seeds of high genetic quality to begin the restoration process (Thomas *et al.* 2014; Basey *et al.* 2015; Nevill *et al.* 2016; Jalonen *et al.* 2018). This step is crucial for the success of restoration, enabling ecosystems to shift from a degraded state to a trajectory that allows species to adapt, persist and evolve over time (Gann *et al.* 2019; Erickson and Halford 2020; Zeng and Fischer 2021). In that regard, obtaining seeds to re-establish plant communities presents an intrinsic challenge for efficient restoration strategies (Bucharova *et al.* 2019). Additionally, determining the optimal distance for relocating them from their original populations to targeted restoration areas is another key issue that has been extensively debated in the literature (Krauss *et al.* 2013; Jorgensen *et al.* 2016; Giencke *et al.* 2018; Nagamitsu and Shuri 2021). The inappropriate use of seed sources can reduce the fitness of populations and compromise the viability of restored ecosystems (Weeks *et al.* 2011; Hufford *et al.* 2012). Therefore, the selection of appropriate seed sources is crucial for ecosystem restoration, as it can influence the outcomes of restoration efforts in both the short and long term (Woolridge *et al.* 2023). In this context, several strategies have been proposed to identify seed sources, for example: (i) the use of seeds of local origin, focussing on preserving potential local adaptations in the species (Hancock and Hughes *et al.* 2014; Breed *et al.* 2018); (ii) the mix of local provenances, emphasizing the balance between the value of local adaptation and the potential for future adaptation (Bucharova *et al.* 2019) and (iii) predictive provenancing, which addresses the selection of source material with the aim of matching or anticipating climate change (Woolridge *et al.* 2023).

In this context, studies of natural populations aimed at elucidating the diversity and genetic structure of species recommended for restoration are crucial for planning seed dispersal strategies across the landscape (Mijangos *et al.* 2015; Van Rossum *et al.* 2022; Broadhurst *et al.* 2023). In general, studies have shown that the patterns of diversity and genetic differentiation in tropical tree populations are influenced by historical processes, environmental changes, the ecology and reproductive systems of the species, and the level of geographic isolation (Bradburd *et al.* 2013; Bucharova *et al.* 2019; Santana *et al.* 2023; Santos *et al.* 2023). Thus, species with a wide geographic distribution, which are found in various environmental conditions and are present in human-modified landscapes, can undergo gradual changes in composition and allelic frequencies. These changes can lead to populations with varying degrees of diversity and genetic differentiation (Whitlock 2008; Boshier *et al.* 2015; Santana *et al.* 2023; Santos *et al.* 2023). Our objective is to elucidate patterns of diversity and genetic structure in natural populations of a tropical tree known as Vinhático (*Plathymenia reticulata*). This endeavour aims to identify appropriate strategies for transferring seeds between source populations and ecosystems undergoing restoration.

We chose *P. reticulata* because this species has been used in restoration projects in the Atlantic Forest and Brazilian Savanna (Ribeiro *et al.* 2018), in addition to having significant potential for logging (Almeida *et al.* 1998; Sambuichi *et al.* 2012). The few studies on this species have been conducted primarily in the Savanna and Atlantic Forest populations, mostly in the Southeast region of Brazil. These studies highlight morphological, physiological and genetic differences, suggesting the existence of Savanna and Forest ecotypes. This underscores the need for a better understanding of the botanical classification of the genus (Lemos *et al.* 2008; Muniz *et al.* 2022, 2023). However, to the best of our knowledge, no genetic study has been conducted on this species in Northeast Brazil for the purpose of supporting ecosystem restoration. Thus, our objectives were (i) to understand the level of diversity and the degree of genetic differentiation among *P. reticulata* populations located in Savanna and Atlantic Forest areas in Northeast Brazil, and (ii) to determine whether environmental, climatic and spatial distances influence the distribution patterns of the gene pool of these populations, with the aim of informing ecosystem restoration efforts.

## Material and Methods

### Characteristics of Plathymenia reticulata

**Figure 1.**
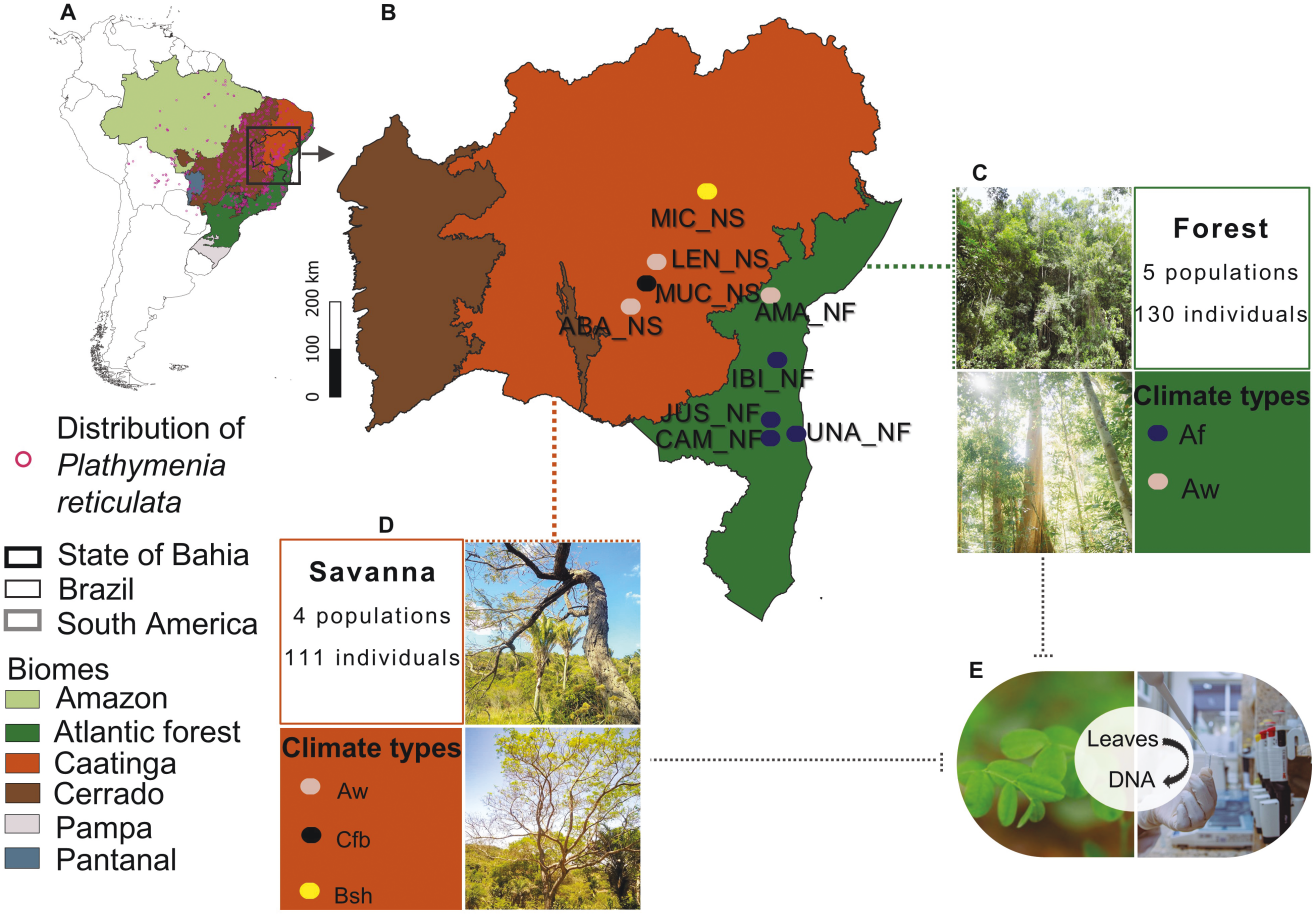
Map of South America, highlighting Brazil, Bahia and their biomes. The geographic distribution of *Plathymenia reticulata is indicated* by purple circles (A). Geographic location, phytophysiognomy and climate types of the sampled populations of *Plathymenia reticulata.* Where: CAM_NF = Camacan, IBI_NF = Ibirapitanga, JUS_NF = Jussari, UNA_NF = Una, AMA_NF = Amargosa, ABA_NS = Abaíra, LEN_NS = Lençóis, MIG_NS = Miguel Calmon and MUC_NS = Mucugê. NF = Native Forest and NS = Native Savanna (B). In brown, the Savanna phytophysiognomy (including those of the Caatinga and Cerrado), and in green, Forest phytophysiognomy (within the Atlantic Forest Biome). Ellipses represent the climate types Af (blue), Aw (beige), Cfb (black) and Bsh (yellow) according to Köppen’s classification for Brazil (Alvares *et al.*, 2013). (C) and (D) represent the sampling schemes and photos of areas, respectively, in Forest and Savanna, along with the climatic types sampled within both physiognomies. (E) Healthy leaves collected from *Plathymenia reticulata* were subjected to laboratory procedures for DNA extraction and amplification using nuclear and chloroplast microsatellite markers. For colour figure refer online version.

This species is a tree native to South America (Warwick and Lewis 2003; GBIF 2023) (Fig 1). It is found in two Brazilian biodiversity hotspots (Myers *et al.* 2000), the Atlantic Forest and the Savanna (including areas of ‘Cerrado’ and ‘Caatinga’). *Plathymenia reticulata* belongs to the Fabaceae family [see Supporting Information**—**Fig. S1] and the subfamily Mimosoideae. It is a monoecious species with polygamous flowers that are pollinated by bees and small insects (Warwick and Lewis 2003; Carvalho 2009). It has high ecophysiological diversity, enabling its broad geographic distribution across gradients of altitude, latitude, precipitation, temperature and soil types (Lemos *et al.* 2008; Carvalho 2009).

### Study areas and sampling

Nine natural populations of *P. reticulata* were sampled across nine municipalities in Bahia State, Brazil (Fig. 1A and B, Table 1), specifically: Camacan (CAM_NF), Ibirapitanga (IBI_NF), Jussari (JUS_NF), Una (UNA_NF), Amargosa (AMA_NF), Abaíra (ABA_NS), Lençóis (LEN_NS), Miguel Calmon (MIG_NS) and Mucugê (MUC_NS). The populations were divided into two categories: ‘Forest’ (NF), characterized by the presence of a canopy (Fig. 1C), and ‘Savanna’ (NS), an open formation with trees and/or shrubs forming a discontinuous layer (Fig. 1D) (Coutinho 2016). The physiognomy of the forest was sampled in populations within the Atlantic Forest Biome (CAM_NF, IBI_NF, JUS_NF, UNA_NF and AMA_NF). On the other hand, samples from the Savanna were collected from populations within the phytogeographic domain of the ‘Caatinga’ (MIC_NS) and transitional mosaics of ‘Caatinga and Cerrado’ (LEN_NS, MUC_NS and ABA_NS) (Harley 1995; Funch *et al.* 2009; Neves *et al.* 2016). Furthermore, to test climate classification as a criterion for seed translocation, we sampled trees in four climate types (‘Af’, ‘Aw’, ‘Cfb’, and ‘Bsh’) using the Köppen classification for Brazil (Alvares *et al.* 2013) [Fig. 1B; **see Supporting Information—****Fig. S2**]. In the tropical climate zone, we sampled the ‘Af’ and ‘Aw’ climate types, characterized, respectively, by the absence of a dry season and a dry season in winter. Regarding the climate type, we sampled the ‘Cfb’ zone, which belongs to the temperate climate zone and features mild temperatures throughout the year. In the semiarid climate zone, we collected samples in a ‘Bsh’ area, an enclave characterized by scarce rainfall in the Brazilian tropical region (Alvares *et al.* 2013).

**Table 1.** Genetic parameters and insertion phytophysiognomies for nine *P. reticulata* populations based on nuclear microsatellite markers (nSSR).

Population	*N*	Phyto	*N*_*a*_	*A*_*R*_	*N*_*e*_	*I*	*A*_*p*_	*H*_*E*_	*H*_*O*_	*F*_*IS*_
CAM_NF	24	Forest	5.89	5.05	2.94	1.25	1.11	0.61	0.45	0.28
IBI_NF	23	Forest	4.22	3.90	2.39	0.96	0.44	0.50	0.51	0.02
JUS_NF	24	Forest	4.11	3.63	2.64	0.93	0.00	0.49	0.31	0.38
UNA_NF	27	Forest	4.78	4.02	2.29	0.95	0.33	0.49	0.45	0.11
AMA_NF	23	Forest	4.89	4.34	2.44	0.98	0.56	0.49	0.47	0.05
ABA_NS	30	Savanna	6.11	5.28	3.66	1.31	0.56	0.62	0.61	0.04
LEN_NS	26	Savanna	5.67	4.95	3.20	1.19	0.22	0.57	0.60	-0.03
MIG_NS	28	Savanna	6.22	5.33	3.50	1.30	0.56	0.62	0.68	-0.08
MUC_NS	28	Savanna	4.89	4.58	3.55	1.18	0.11	0.58	0.58	0.03
** Mean**	**25.89**	**–**	**5.20**	**4.56**	**2.96**	**1.12**	**0.43**	**0.55**	**0.52**	**0.09**

Phyto, phytophysiognomy; *N*_*a*_, number of alleles per locus; *A*_*R*_, allelic richness; *Ne*, effective number of alleles per locus; *I*, Shannon index; *Ap*, number of private alleles; *H*_*E*_, expected heterozygosity; *H*_*O*_, observed heterozygosity; *F*_*IS*_, fixation index; CAM_NF, Camacan; IBI_NF, Ibirapitanga; JUS_NF, Jussarí; UNA_NF, Una; AMA_NF, Amargosa; ABA_NS, Abaíra; LEN_NS, Lençóis; MIG_NS, Miguel Calmon and MUC_NS, Mucugê. NF, Native Forest and NS = Native Savanna. Note: Underlined values indicate populations with genetic parameters below the general average.

In each population, an average of ~26 adult individuals was sampled, using the circumference at breast height ≥15 cm and a minimum distance of 100 m between individuals as inclusion criteria for all populations. This criterion was used to sample a genetic pool representative of the population while simultaneously avoiding the sampling of genetically related individuals (Oliveira 2012). However, for the CAM_NF and JUS_NF populations, where sampling was conducted through a census in 400 × 400 m plots from a previous study (Oliveira 2012), we selected the 24 individuals who were the most geographically distant from each other. All individuals were georeferenced, and young leaves were collected for the extraction of total genomic DNA.

### Laboratory procedures

We followed the Doyle and Doyle (1987) extraction protocol, which involved macerating the samples in a Precellys® Evolution using a 2 % CTAB buffer, with approximately eight leaflets per sample. The DNA extracted from the samples was quantified using a 1 % agarose gel, by comparing the samples to dilutions of a known standard concentration of bacteriophage *λ* DNA. Afterward, the 233 samples collected from the nine *P. reticulata* populations were genotyped using nine nuclear microsatellite loci (nSSRs—Simple Sequence Repeats) (Oliveira *et al.* 2012) and three chloroplast microsatellite loci (cpSSR2, cpSSR5 and cpSSR10) (Weising and Gardner 1999). Polymerase chain reactions were conducted according to the specifications described by Oliveira *et al.* (2012) and Weising and Gardner (1999). Subsequently, the electrophoresis of the fragments was performed using an ABI 3500 genetic analyser (Applied Biosystems, USA). For this purpose, 2 µL of the PCR product mix (multiload system), 0.3 µL of GeneScan™, 500 µL of Liz™ (Applied Biosystems, Thermo Fisher Scientific, Inc., Waltham, MA, USA) and 7.7 µL of formamide (Applied Biosystems) were used. The analysis of electropherograms for genotyping was performed using GeneMarker software (version 1.95) (SoftGenetics, State College, PA, USA). Data verification and testing for null alleles were performed using Micro-Checker 2.2.3 (Van Oosterhout *et al.* 2004).

### Genetic analysis with nSSR and cpSSR molecular markers

The following population genetic parameters were calculated using GenAlEx (Peakall and Smouse 2006): number of alleles per locus (*Na*), effective number of alleles per locus (*Ne*), number of private alleles (*Ap*), Shannon Index (*I*) and historical gene flow (*Nm*). While allelic richness (*A*_*R*_), observed (*H*_*O*_) and expected (*H*_*E*_) heterozygosity and Wright’s *F*-statistics: *F*_*ST*_ values for paired populations and the fixation index (*F*_*IS*_), calculated using the random model from Weir (1996), were determined with the FSTAT software (Goudet 1995). Then, the decomposition of total genetic variation into hierarchical levels was assessed through two analyses of molecular variance (AMOVA), conducted using the Arlequin (v.3.5) software (Excoffier and Lischer 2010). In the first AMOVA, we consider the decomposition of total genetic variation into hierarchical levels based on the source of variation: phytophysiognomy (among phytophysiognomies, populations within phytophysiognomies and individuals within populations). In the second AMOVA, the sources of variation were climate typology (among climate, populations within climate and individuals within populations).

To assess the genetic structure among the nine sampled populations, we used three complementary methods: Wright’s *F*-statistic (*F*_*ST*_), Bayesian simulation and multivariate analysis. The Bayesian simulation was carried out using STRUCTURE 2.3.4 (Pritchard *et al.* 2000), with one to ten clusters (*K* = 1–10) and 10 runs for each *K*. The simulation had a running period of 10 000 and 10 000 replications. Then, the most likely *K* value was determined using the Δ*K* method with the Structure Harvester software (Earl and Vonholdt 2012). Multivariate analysis was conducted using discriminant analysis of principal components (DAPC) with the Adegenet package in R (Jombart and Ahmed 2011). The *find.clusters* function was used to determine the number of clusters. Then, to describe the relationships between the clusters identified in the nine *P. reticulata* populations sampled, we used the generic DAPC function.

To assess the influence of edaphic, bioclimatic and spatial distance variables on the genetic differentiation of *P. reticulata* populations, two methods were used: (i) Mantel tests to evaluate the effects of isolation by distance and isolation by environment, and (ii) distance-based redundancy analysis (*dbRDA*). For Mantel tests, we calculated the paired spatial distance between populations using the geographic coordinates of the collection points in the QGIS software. Subsequently, the coordinates were used to extract the following edaphic variables: bulk density of the fine earth fraction (*bdod*), soil pH (*phh2o*), soil organic carbon content (*soc*), organic carbon density (*ocd*), proportion of sand particles (*sand*) and soil cation exchange capacity *(cec*) (Poggio *et al.* 2021). The variables were downloaded for the soil layer between 15 and 30 cm depth in a Raster file with a spatial resolution of 1 km (https://files.isric.org/soilgrids/latest/data/). As bioclimatic predictors for the collection points, we extracted the 19 bioclimatic variables from the WorldClim2 dataset, which has a spatial resolution of 30 s (https://www.worldclim.org/data/bioclim.html). Next, we assessed the correlation between soil variables and bioclimatic variables separately using the variance inflation factor (*VIF*), employing the vif.cca function from the Vegan package in R. For this analysis, we excluded variables with *VIF* values >5, indicating strong collinearity (Zuur *et al.* 2009). After removing the collinear variables, all subsequent analyses were based on *BIO2* (Mean Diurnal Range), *BIO12* (Annual Precipitation), *cec, soc* and *phh2o* [**see Supporting Information—**Figs. S3 and S4]. After this step, to calculate the Mahalanobis distances for the edaphic and bioclimatic variables separately, we used the D2.dist function from the biotools package in the R environment. Followed by the execution of simple Mantel tests using the vegan package. As a measure of genetic distance, we used *F*_*ST*_ values. In contrast, we considered edaphic and bioclimatic variables separately as environmental distances (i.e. the previously calculated Mahalanobis distances). Simple Mantel tests were conducted to compare genetic and spatial distances, as well as genetic distances with edaphic or bioclimatic factors.

To assess the association between each of the edaphic and bioclimatic variables and the degree of genetic divergence among populations, we performed a distance-based redundancy analysis (*dbRDA*) (Legendre and Legendre 2014). This approach has been used in plant and animal genetics and genomics studies to assess how environmental characteristics influence the distribution of the gene pool across the landscapes under study (Muniz *et al.* 2022). This is a multivariate method that combines ordination with a multivariate regression approach to identify linear relationships between a matrix of explanatory variables and a matrix of response variables. This method effectively identifies linear combinations of the response variable, namely, the genetic structure among populations, and multivariate predictors, which, in this case, correspond to soil, bioclimatic and spatial variables (Legendre and Legendre 2014). For this purpose, to measure genetic structure, we applied genetic spatial principal component analysis (*sPCA*) scores obtained using the *spca* function from the Adegenet package (Jombart 2008). In this analysis, we selected only the scores from the first two axes of the *sPCA* [**see Supporting Information—**Fig. S5], which summarize the genetic variability of the evaluated individuals, while controlling for spatial autocorrelation among them using Moran’s *I* statistic (Jombart 2008). Finally, we used Moran’s spatial eigenvectors (*MEM*) as spatial predictors, based on the latitude and longitude of *P. reticulata* populations, using the spdep and adespatial packages in the R software. *MEMs* are derived from the ordering (*PCoA*) of a truncated matrix, which is based on the geographic locations of localities using Euclidean distance. This process generates eigenvalues that are identical to Moran’s *I* spatial correlation coefficients (Dray *et al.* 2012). In this analysis, only *MEM1* and *MEM3* were significant, with *R*² values of 0.65 and 0.15, respectively. Therefore, given that *MEM1* accounted for the greatest spatial variation, we chose this eigenvector as our spatial predictor. To select the optimal *dbRDA* model, we conducted forward variable selection using the ordiR2step function from the Vegan package (Oksanen *et al.* 2020). The stopping criteria were as follows: a variable with a significance level of *P* < 0.05, 1,000 permutations and the adjusted *R*^2^ of the overall model. Finally, we estimated the proportion of variance explained by each variable (partial *R*^2^) using the varpart function in the vegan package (Oksanen *et al.* 2020).

We conducted complementary analyses using cpSSR loci with the Haplotype Analysis software (version 1.04) (Eliades and Eliades 2009). We calculated the number of haplotypes (*N*_*H*_), number of effective haplotypes (*H*_*Ne*_), private haplotypes (*P*_*H*_), haplotype richness (*H*), haplotype diversity (*H*_*E*_), mean genetic distance between individuals (*D*^*2*^*sh*) and the measure of population subdivision (*F*_*ST*_). To test the hypothesis of isolation by distance between pairs of populations, the Simple Mantel test was conducted in the R environment, using the Vegan package and Pearson’s correlation coefficient.

## Results

### Diversity and structure based on nSSR markers

**Figure 2.**
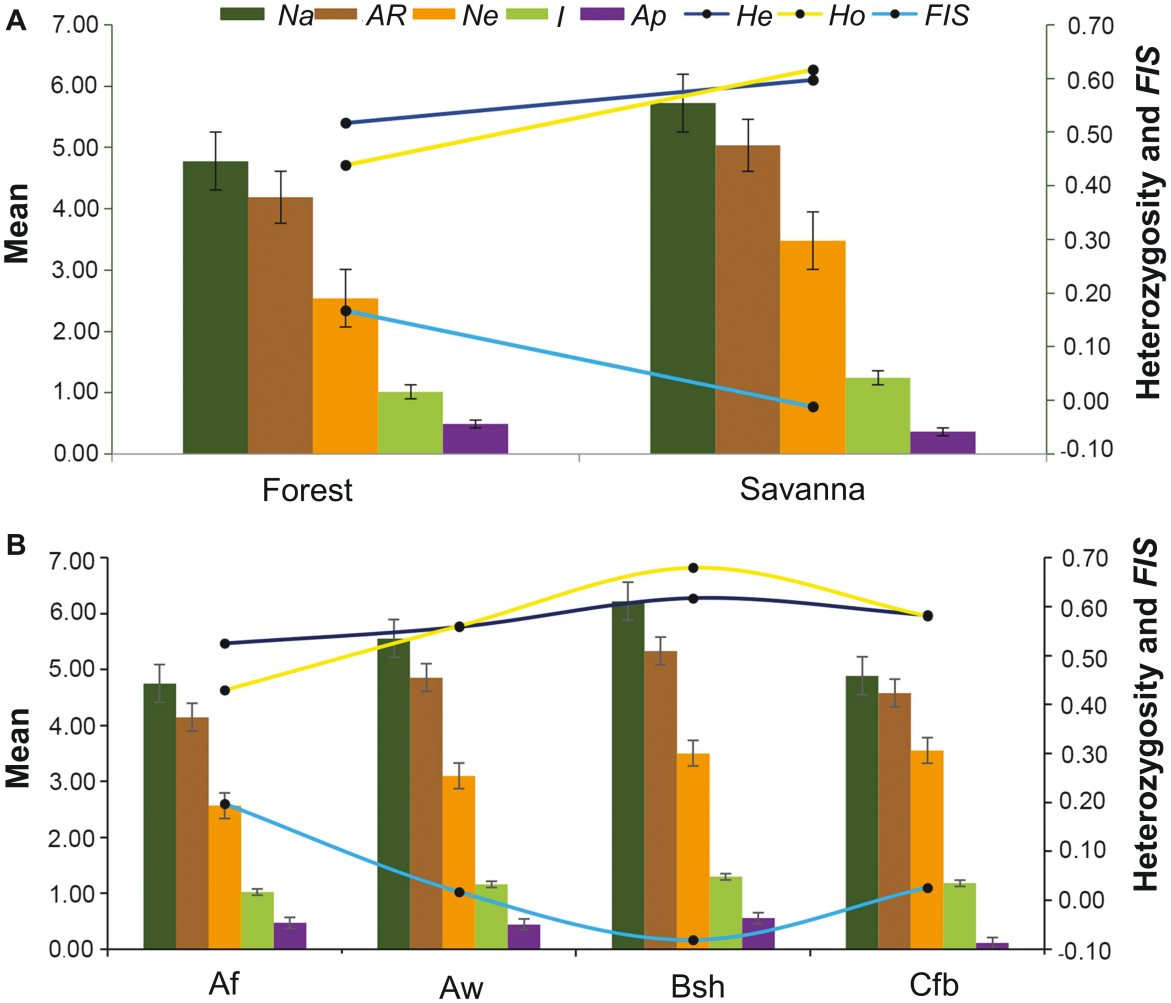
Genetic diversity parameters, including standard error bars, for *P. reticulata* across different phytophysiognomies (Forest or Savanna) (A) and climate types (B). *Na*, number of alleles per locus; *A*_*R*_, allelic richness; *Ne*, effective number of alleles per locus; *I*, Shannon index; *Ap*, number of private alleles; *H*_*E*_, expected heterozygosity; *H*_*O*_, observed heterozygosity; *F*_*IS*_, fixation index.

The nine populations of *P. reticulata* evaluated in the state of Bahia, Brazil showed great variation for all genetic parameters (Table 1). Overall, the populations within the Forest phytophysiognomy (CAM_NF, IBI_NF, JUS_NF, AMA_NF and UNA_NF) showed lower values than the average for six of the eight genetic parameters evaluated, except for Ap and *F*_*IS*_ (Table 1). In contrast, the populations within the Savanna phytophysiognomy (ABA_NS, LEN_NS, MIG_NS and MUC_NS) generally showed values above the average for six of the eight genetic parameters, along with lower *F*_*IS*_ values. This result revealed a clear pattern of increased global diversity (i.e. higher *Na*, *A*_*R*_, *Ne*, *I*, *H*_*E*_ and *H*_*O*_) and a lower fixation index (*F*_*IS*_ in Savanna compared to forest areas (Fig. 2A)). When the populations were grouped according to climatic typologies, the Bsh climate typically showed the highest genetic diversity (*Na*, *A*_*R*_, *I*, *Ap*, *H*_*E*_, *H*_*O*_) and the lowest *F*_*IS*_, in contrast to the Af climate, which had the lowest diversity and the highest *F*_*IS*_ (Fig. 2B).

The decomposition analysis of total genetic variation into hierarchical levels showed that genetic variability was significantly structured across different vegetation types (*F*_*CT*_ = 0.402, *P*-value < 0.05), with 40.28 % of the genetic variability attributed to differences between Savanna and Forest stands (**see Supporting Information—**Table S1). The variation between climate types was not significant, accounting for only 12.10 % of the variation observed at the hierarchical levels both between and within populations. In both hierarchical analyses, the majority (> 50 %) of molecular variation was observed within the populations studied (*F*_*ST*_ = 0.494, *F*_*ST*_* *= 0.404, *P*-value < 0.05).

When assessing historical gene flow, the average number of migrants per generation (*N*_*m*_) was 5.8 for Savanna populations and 5.2 for forest populations. However, the average gene flow (*N*_*m*_) between Savanna and Forest populations was only 0.7. Finally, populations from the Aw/Bsh groups showed greater gene flow compared to other combinations of climatic typologies, in contrast to the low gene flow observed among populations from the Af, Bsh and Cfb classifications (*N*_*m*_ = 0.7).

**Figure 3.**
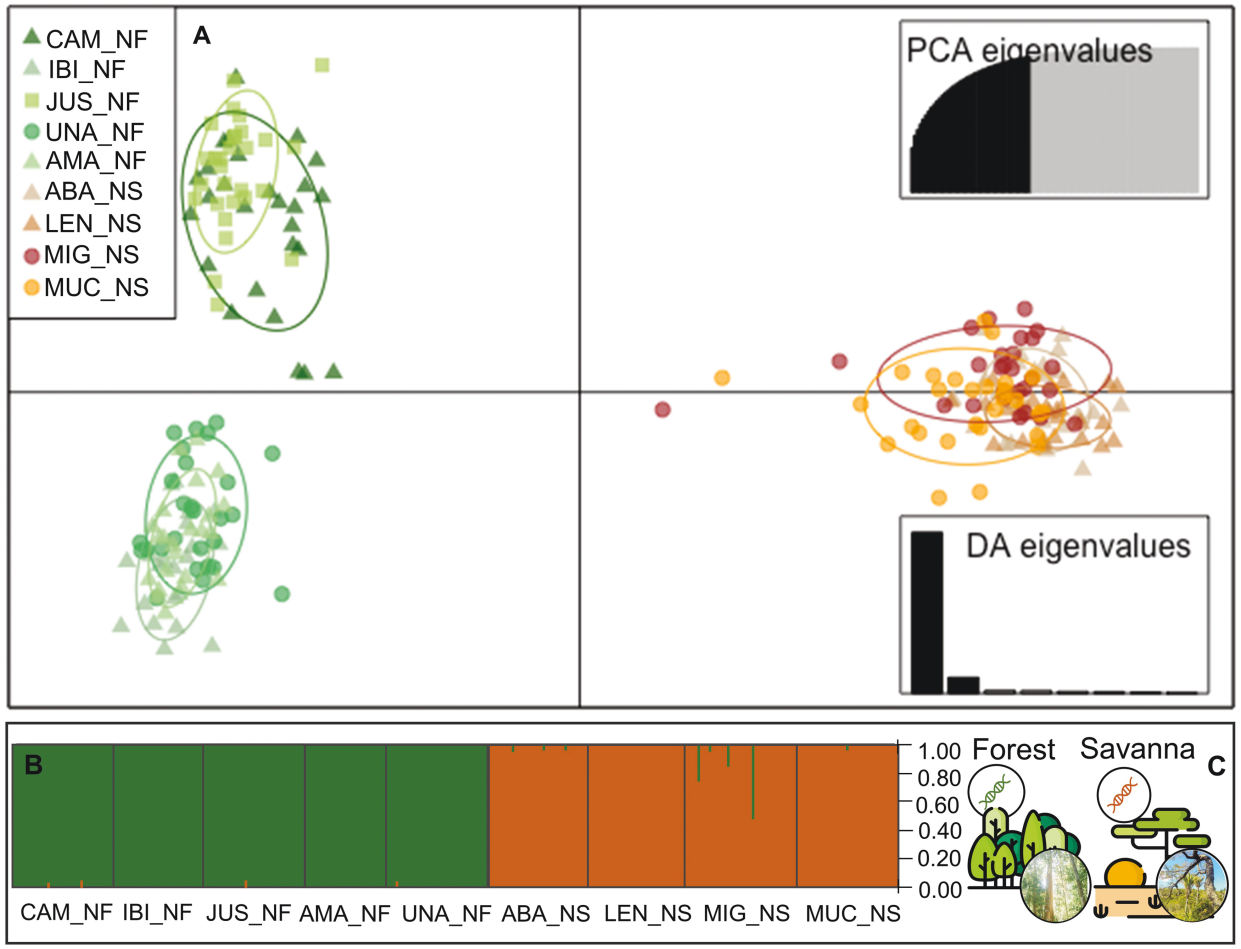
Genetic differentiation among nine populations of *P. reticulata* based on nuclear microsatellite markers (nSSR). (A) DAPC. (B) Structure bar plot (*K* = 2). Each sampled individual of *P. reticulata* is represented by a single vertical line, with different colours (green or orange) indicating the assignment probabilities to the two inferred genetic clusters. (C) Schematic representation of the gene pools observed (*K* = 2), grouping the samples according to their classification into Savanna (Orange) and Forest (Green) categories. For colour figure refer online version.

In the genetic structure analysis of the nine populations, an average *F*_*ST*_ value of 0.282 was obtained, indicating a strong genetic structure. Very high *F*_*ST*_ values were observed among populations located in different phytophysiognomies (Savanna versus Forest, shown in dark blue in the lower left, **see Supporting Information—**Fig. S6). The lowest *F*_*ST*_ values were reported among Savanna populations, while low, moderate and high values were identified among Forest populations [**see Supporting Information—**Fig. S6]. The DAPC grouped *P. reticulata* individuals into genetically related groups corresponding to the Forest and Savanna typologies (Fig. 3A). The results also indicated that the Forest gene pool consists of two subgroups: Forest I, which includes the JUS_NF and CAM_NF populations, and Forest II, comprising the IBI_NF, UNA_NF and AMA_NF populations. Furthermore, the Bayesian analysis conducted on the structure indicated that the most probable number of *K* was 2 (Fig. 3B), demonstrating that individuals from the nine populations were divided into two gene pools. The first group consisted of the Forest populations, while the second consisted of the Savanna populations.

Simple Mantel tests revealed a significant correlation between environmental (edaphic and bioclimatic) factors and geographic matrices and genetic distance [**see Supporting Information—**Table S2]. The highest correlation observed (0.78) was found in the association between climate and genetic differentiation. This indicates that environmental heterogeneity and spatial scale together influence the genetic structure of native *P. reticulata* populations [**see Supporting Information—**Table S2].

**Figure 4.**
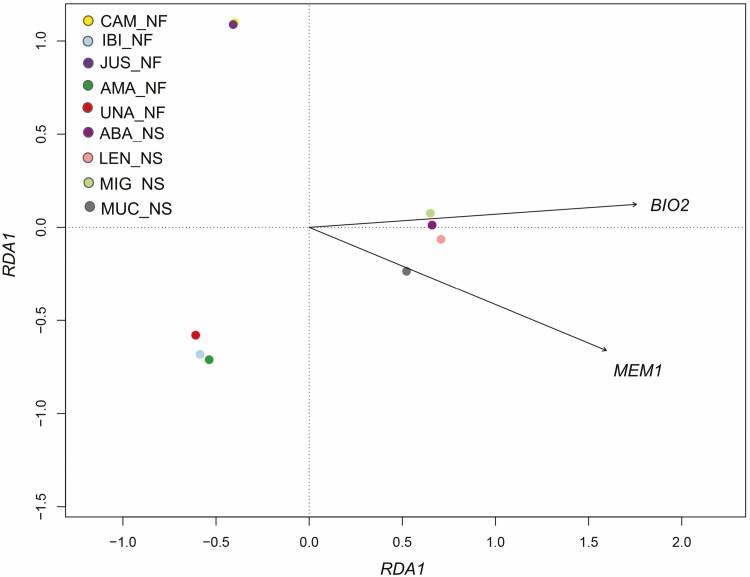
Genetic differentiation among nine *P. reticulata* populations in relation to *MEM1* and *BIO2*, as determined by distance-based redundancy analysis (*dbRDA*). Evidencing *K* = 2, with one grouping among populations in Savanna phytophysiognomy (ABA_NS, LEN_NS, MIG_NS and MUC_NS) and the other grouping formed among populations in Forest (composed of two subgroups). *BIO2*, Average Daily Temperature Range (maximum temperature—minimum temperature), *MEM1*, predictor of spatial variation.

The *sPCA* test revealed a global spatial genetic structure among *P. reticulata* populations, as demonstrated by the first two axes [**see Supporting Information—**Fig. S5], which were visualized in geographic space [**see Supporting Information—**Fig. S7]. The *dbRDA* analysis, which was used to identify the variables associated with genetic divergence, showed a highly significant association of population genetic divergence with *MEM1* (a spatial predictor) and *BIO2* (Mean Diurnal Temperature Interval, **see Supporting Information—**Table S3). Both variables are related to the RDA1 axis, which significantly explained the genetic variation [**see Supporting Information—**Table S4, Fig. 4] and grouped the populations found in Savanna areas, while the populations in Forest were composed of two subgroups (Fig. 4). The partitioned analysis of variance revealed that *BIO2* had a greater explanatory power compared to *MEM1*, although the combined effects of these variables accounted for 51 % of the genetic structure [**see Supporting Information—**Fig. S8].

### Haplotype diversity and structure based on cpSSR

**Figure 5.**
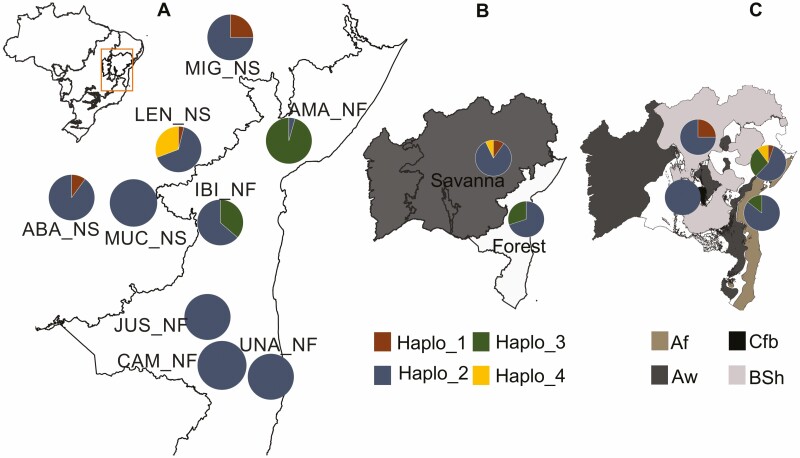
Map of Brazil, highlighting the state of Bahia and showing the (A) geographic distribution of the four haplotypes across the population. (B) Distribution of haplotypes across different phytophysiognomies (Forest or Savanna). (C) Distribution of haplotypes across different climate types. Each haplotype (Haplo) is identified by a distinct colour as indicated in the legend. For colour figure refer online version.

In the nine populations, four haplotypes were observed, with 72.3 % of individuals carrying the most frequent haplotype (haplo_2) [Fig. 5A, **see Supporting Information—**Table S5]. Additionally, one haplotype (haplo_3) in the Forest populations was shared between IBI_NF and AMA_NF (Fig. 5A and **see Supporting Information—**Table S5). While populations were categorized into vegetation types, Forest and Savanna, two haplotypes (haplo_1 and haplo_4) were found exclusively in populations from the Savanna category (present in all Savanna populations except for MUC_NS) [Fig. 5B, **see Supporting Information—**Table S5]. Given the climate type, the presence of four haplotypes in the Aw region is notable, with one of them (Haplo_4) being exclusive [Fig. 5C, **see Supporting Information—**Table S5].

The populations with the highest numbers of haplotypes (*N*_*H*_), effective haplotypes (*H*_*Ne*_) and haplotype richness (*H*) were LEN_NS, IBI_NF and MIG_NS. The highest haplotypic diversity (*H*_*E*_) was observed in LEN_NS (0.50), IBI_NF (0.48) and MIG_NS (0.38), with low overall averages across populations (0.18) [**see Supporting Information—**Table S6]. When considering phytophysiognomy, the Savanna group showed higher *N*_*H*_, *H*_*Ne*_, *P*_*H*_, *H* and average genetic distance between individuals (D2sh), as well as lower *HE*, compared to the Forest group [**see Supporting Information—**Table S7]. In the Aw climate classification, which includes the AMA_NF, ABA_NS and LEN_NS populations, the highest values of *N*_*H*_, *H*_*Ne*_, *P*_*H*_, *H* and D2sh were observed [**see Supporting Information—**Table S7].

Genetic structure analysis revealed an overall *F*_*ST*_ of 0.554, with the greatest differentiation observed in comparisons involving AMA_NF with CAM_NF, JUS_NF, UNA_NF and MUC_NS [**see Supporting Information—**Table S8]. There was no correlation between genetic and geographic distances (*P*-value > 0.05), suggesting that isolation by distance may not have influenced the genetic differentiation observed in *P. reticulata* according to cpSSR data [**see Supporting Information—**Fig. S9].

## Discussion

Repopulating areas with seeds and seedlings is a strategy aimed at facilitating the restoration process in places that have lost resilience and/or are far from fragments capable of providing seeds through natural dispersal. Introducing them into degraded areas in such instances not only lays the foundation for species diversity and the restoration of forest strata but also contributes to restoring biodiversity at the genetic level (Kettenring and Tarsa 2020). Here, we describe the diversity and genetic structure of nine native *P. reticulata* populations that can serve as seed sources, acting as gene repositories for newly restored populations.

### Genetic diversity based on nSSR: vegetation typology and the biology of P. reticulata are likely the main factors influencing the species’ genetic diversity

Overall, the populations of *P. reticulata* evaluated showed low to moderate genetic variability compared to nine other tree species living in anthropogenic landscapes of the Brazilian Atlantic Forest (Santana *et al.* 2023). This result may be attributed to the impact of human activities (such as deforestation and logging), in conjunction with the reproductive system of the species. Considering that *P. reticulata* is a polygamous plant (i.e. it has unisexual and bisexual flowers), it is possible for a rate of self-fertilization to occur, especially in anthropized landscapes. This could favour the occurrence of inbreeding, leading to a reduction in genetic diversity in populations (Almeida *et al.* 1998; Warwick and Lewis 2003; Santana *et al.* 2023).

Particularly, the Atlantic Forest populations showed lower levels of diversity compared to those in the Savanna. Our hypothesis is that this difference is due to the environmental similarity between the species’ centre of genetic diversity and the Savanna populations sampled in this study. There is evidence that the centre of origin and genetic diversity of the species is the Central Brazilian Savanna (Novaes *et al.* 2010). This could facilitate easier colonization by the species and result in greater genetic diversity among the Savanna populations evaluated in this study. On the other hand, since the environmental conditions of the Atlantic Forest greatly differ from those of the Savanna, the Atlantic Forest could represent the peripheral distribution zone for the species. Here, only a few individuals might be able to colonize this new habitat, resulting in lower genetic diversity within these populations. Over evolutionary time, the observed differences in genetic diversity and composition, as seen in the analysis of genetic structure and influenced by the environmental variation of these phytophysiognomies, could have led to the reported morphological differentiation in *P. reticulata* (Lemos *et al.* 2008). These findings support the existence of one ecotype for the Atlantic Forest and another for the Savanna, as demonstrated in phenotypic tests of *P. reticulata* in common gardens (Lemos *et al.* 2008).

Another hypothesis that supports the understanding of the genetic diversity levels found among the vegetation types is the vertical and horizontal structure of the two vegetation formations. It is possible that open areas such as the Savanna may exhibit greater anemochorous seed dispersal distances and cross-pollination rates, as the characteristics of the landscape can promote higher wind speeds, leading to increased seed dispersal distances and facilitating longer flights of small pollinating insects. Thus, since *P. reticulata* is a species with anemochorous seeds and relies on bees and small insects for pollination of its flowers (Warwick and Lewis 2003; Carvalho 2009), a higher gene flow within and between the populations that constitute the Savanna group would be expected, in comparison to the Forest populations. This could explain the observed pattern of genetic diversity. This hypothesis is supported by the higher number of migrants and the less pronounced genetic structure among Savanna populations compared to Forest populations, as evidenced in our analyses.

Finally, it is also important to highlight that populations in the Atlantic Forest showed the highest values of the fixation index (*F*_*IS*_), a particularly concerning result for CAM_NF (*F*_*IS*_ = 0.28) and JUS_NF (*F*_*IS*_ = 0.38), indicating an excess of homozygotes. This result may be attributed to the sampling design adopted for these populations, which, unlike the others, consisted of a census in the plots and involved sampling all individuals for genetic analyses (Oliveira 2012). This could potentially explain the high *F*_*IS*_ values, as we observed the presence of spatial genetic structure in the spatial principal component analysis (*sPCA*), which supports a previous study on *P. reticulata* that identified genetic structure up to 125 m (Oliveira 2012). Therefore, since allele sampling also relies on spatial genetic structure (Kashimshetty *et al.* 2017), the physical distance between *P. reticulata* parent trees should be considered during seed collection. Therefore, the number and distance of parent trees sampled in source populations strongly influence the representativeness of the *P. reticulata* gene pool in areas under restoration (Hoban and Schlarbaum 2014; Hoban and Strand 2015). In this context, the strategy used for collecting seeds within a population can reduce the risks of inbreeding and increase the genetic diversity of the batches prepared for restoration.

### Considering restoration based on the allele composition of P. reticulata populations

Ecosystem restoration has progressively evolved in terms of objectives, strategies and techniques, with the incorporation of genetic approaches offering new perspectives for restoration across the three levels of biodiversity (genetic, species and ecosystems). In the present study, based on the distribution patterns of genetic diversity of *P. reticulata* reported and the possible modulating forces previously discussed, we argue that the genetic parameters (Table 1, Fig. 2) can be used to propose efficient strategies for restoring ecosystems in Atlantic Forest and Savanna areas. In particular, based on the number of effective alleles, *A*_*R*_, and expected heterozygosity, which revealed low to moderate genetic diversity in natural populations, we propose that it would be ideal to collect seeds from different source populations to compose lots destined for restoration. However, it is important to note that the source populations for each batch must be located within the same biome (further details will be provided in Section 4.3).

Collecting from multiple sources would be particularly relevant in populations with low genetic diversity, aiming to expand the genetic base of seed lots and, consequently, the genetic diversity of the restored areas. In this context, although collecting seeds from multiple populations does not necessarily ensure equitable representation in restored areas (Kucera *et al.* 2022), this strategy increases the likelihood of sampling rare and private alleles, thereby promoting genetic diversity.

Based on the levels of diversity reported in this study, our recommendation to use multiple seed sources aligns with a recent global synthesis. This synthesis emphasized that mixing propagules from different local populations is the best approach to increaseing genetic diversity in restored populations (Wei *et al.* 2023). Maximizing genetic diversity in populations is an excellent strategy to increase the probability of long-term restoration success since genetic diversity is the raw material upon which natural selection works (Schäfer *et al.* 2020; Van Der Merwe *et al.* 2021; Broadhurst *et al.* 2023). In this context, we expect greater genetic diversity to be associated with a stronger adaptive potential in populations, leading to successful restoration (Gann *et al.* 2019; Axelsson *et al.* 2020; Stange *et al.* 2021). This perspective is particularly important because climate change will necessitate that populations in restored ecosystems be capable of making adaptive adjustments in response to environmental changes (Moreno-Mateos *et al.* 2020; Broadhurst *et al.* 2023).

We also emphasize the importance of focussing on building restored populations with high genetic diversity (Bucharova *et al.* 2019; Fernandes *et al.* 2023; Wei *et al.* 2023). Since genetic diversity is closely linked to the functioning of biological communities and helps to restart ecosystem processes, it supports the evolutionary potential of species (Aavik and Helm 2018; Jalonen *et al.* 2018; Jordan *et al.* 2018, 2019; Axelsson *et al.* 2020). From this perspective, we emphasize that during the process of ecosystem restoration, it is necessary to consider not only the richness and composition of species but also the genetic diversity within the populations of these species (Aavik and Helm 2018; Jordan *et al.* 2019).

It is also important to mention that promoting the composition of seed lots from diverse sources can help minimize potential impacts on the survival of source populations due to excessive collection to meet restoration demand (Meissen *et al.* 2015, 2017). Therefore, the approach suggested in this study becomes even more relevant, as ecosystem degradation reduces the number of remaining areas and, consequently, the availability of seed and allele reservoirs (Santos *et al.* 2021; Lima *et al.* 2024). Thus, by providing information on the population genetics of a tropical tree species in deforested landscapes and suggesting potential restoration strategies, we aim to emphasize the importance of genetic parameters in restoration planning and include genetic diversity, which is often overlooked (Kettenring and Tarsa 2020; Wei *et al.* 2023). Furthermore, the genetic characterization of these populations will enable the monitoring and evaluation of the effectiveness of genetic restoration efforts, as our study offers reference points based on the patterns observed in natural populations of *P. reticulata*. Similar perspectives were addressed in studies that used genetic parameter information in tropical species to evaluate various aspects. These aspects include the effect of using multiple seed batches collected over several years (Zeng and Fischer 2021), the levels of genetic diversity in species within restored areas compared to native populations (Zucchi *et al.* 2018; Cordeiro *et al.* 2019), and the estimation of inbreeding and gene flow between restored and native populations (de Souza *et al.* 2022; Sujii *et al.* 2017, 2021).

### *Mix seed sources, but within the same biome: environmental heterogeneity also* shapes *the genetic structure of P. reticulata*

The ‘local is better’ paradigm is generally associated with the superior performance of locally sourced seeds (Mckay *et al.* 2005). However, its definition is complex (Boshier *et al.* 2015) and involves considering whether geographic proximity, genetic, phylogenetic or ecological similarity should be used to define local provenances (Dupré La Tour *et al.* 2020). Here, we present our findings on how geographic distance and environmental heterogeneity affect the genetic variation among *P. reticulata* populations. This information could be valuable for identifying local sources when relocating seeds of this species for ecosystem restoration.

Our analyses revealed low allelic flow across all populations of *P. reticulata* under study, consistent with the strong genetic structure observed with both sets of markers (nSSR *F*_*ST*_ = 0.282; cpSSR *F*_*ST*_* *= 0.554). When investigating the possible causes of this differentiation in the population’s gene pool, we found that the classification of climatic zones (Aw, Af, Bsh and Cfb) had little influence on the partitioning of variability, and therefore, had limited potential to explain the patterns of diversity and genetic structure. This outcome likely stemmed from confounding factors introduced by using zones as predictors of genetic similarities and divergences. Given that the same climate zone can encompass various vegetation types, for example, the AMA_NF population is in a Forest, while ABA_NS and LEN_NS are in a Savanna. However, all three populations are located in an Aw climate zone.

Unlike climatic classification, environmental classification based on phytophysiognomy greatly influences the genetic differentiation of *P. reticulata* populations (AMOVA, *FST*, DAPC and Structure), indicating that vegetation type is an important driver of the gene pool. Therefore, we believe that phytophysiognomy may act as a barrier to gene flow, leading to the strong genetic structure observed (Sexton *et al.* 2014). Our hypothesis is that *P. reticulata* is undergoing speciation, driven by natural selection due to environmental differences between the Savanna and the Forest. This leads to divergent adaptations, potentially explaining the observed pattern of genetic differentiation. This hypothesis aligns with the findings of genetic and morphological studies on *P. reticulata*, which suggest the existence of one ecotype for the Atlantic Forest and another for the Savanna (Lemos *et al.* 2008; Muniz *et al.* 2022). Furthermore, we observed greater gene flow (*N*_*m*_) between similar environments than between the Savanna and Forest ecosystems. This result is consistent with isolation by environment and may stem from the indirect effect of natural selection on gene flow between contrasting habitats. Selection might favour adaptations to local conditions, potentially reducing the fitness of immigrants in non-local environments and thus imposing barriers to gene flow (Sexton *et al.* 2014). However, future experimental studies are needed to uncover the ecological/microevolutionary mechanisms behind this apparent isolation by environment.

Finally, when we examined the influence of geographic distance on the genetic differentiation of populations, we observed no significant correlation using chloroplast markers. Although this result indicates there is no genetic isolation by distance, caution is necessary, as we used only three cpSSR markers located in a highly conserved genome, such as the chloroplast genome (Shaw *et al.* 2007; Wick *et al.* 2011). On the other hand, geographic distance had an effect on genetic differentiation for nuclear markers, particularly when associated with environmental heterogeneity. This result suggests that relying solely on geographic boundaries to define seed translocation might be an oversimplification that does not align with the patterns of genetic variability observed in *P. reticulata* (Boshier *et al.* 2015). This reinforces the idea that, without also considering the environmental heterogeneity, there may not be a fixed geographical scale to determine how far seeds can be translocated from the source population to restoration areas (Erickson and Halford 2020).

By assessing the influence of environmental heterogeneity, considering both soil conditions and climate variables, on genetic differentiation, we found that soil composition and climatic factors significantly affected the distribution of gene pool found in within *P. reticulata* populations. The Savanna, compared to the Forest, is characterized by low soil fertility and stressful climatic conditions, such as significant temperature variation throughout the day (Maracahipes *et al.* 2018). Thus, survival in these two environments requires distinct adaptive strategies, which could explain the pattern of genetic differentiation between Savanna and Forest populations, which are modulated by these edaphoclimatic variables (Maracahipes *et al.* 2018). Therefore, we emphasize that the translocation of *P. reticulata* seeds from native remnants to restoration sites must consider the classification of Forest versus Savanna and the purchase or collection of seeds should be restricted to the biome where they will be used.

This recommendation aims to establish limits that ensure the safe movement of seeds across the landscape. Given the pattern of genetic structure identified, it is plausible that translocating *P. reticulata* seeds between distinct phytophysiognomies (Savanna versus Forest) affects the success of restoration areas. Because individuals from non-local seeds used in restoration may have a reduced ability to persist in the ecosystem due to poor local adaptation—that is, an inability to adapt to a phytophysiognomy different from their origin (Lemos *et al.* 2008; Muniz *et al.* 2023). Another concern that needs to be considered is that translocating *P. reticulata* seeds between different environments may increase the risk of genetic swamping in local populations (Rutherford *et al.* 2019). Furthermore, the low number of migrants between Forest and Savanna suggests a potential partial reproductive barrier between populations in these environments (Muniz *et al.* 2023). Therefore, using seeds from different environments in restoration could hinder gene flow between the restored and remaining populations. Such a scenario would hinder the promotion of connectivity between restored and native areas, which is crucial for the long-term success of restored areas (Proft *et al.* 2018; Sujii *et al.* 2021).

Finally, another potential consequence of seed translocation from Savanna to Forest and vice versa could be a higher risk of outbreeding depression (loss of fitness when highly genetically distinct populations interbreed, according to Frankham *et al.* 2011; Hufford *et al.* 2012). While inbreeding depression is more common in trees (Edmands 2007; Liddell *et al.* 2021), significant genetic differentiation between populations can signal serious risks of outbreeding depression when genetic pools are mixed (Liddell *et al.* 2021). The likelihood of outbreeding depression could be significant in species found across diverse environments, as demonstrated in the case of *P. reticulata* (Frankham *et al.* 2011). Documented across various taxa, including tree species (Jia *et al.* 2020), outbreeding depression can affect the performance of hybrids in restoration projects (Keller *et al.* 2000; Forrest *et al.* 2011; Goto *et al.* 2011; Hufford *et al.* 2012). Thus, the possibility of outbreeding depression is presented here as a stimulus to consider its risk, as it could affect the long-term fitness and sustainability goals of restored populations (Hufford *et al.* 2012).

We mention these possibilities related to seed sources because restoration involves reassembling communities (Weidlich *et al.* 2021) and failure caused by inappropriate translocations can lead to economic and ecological losses when attempting to re-establish functional communities. In this context, for *P. reticulata*, the concept of local seeds can be associated with the distinction between Savanna and Forest, as our results indicate that this classification reflects the pattern of genetic differentiation in the species. Furthermore, as we previously discussed, we recommend that whenever possible, seeds from different sources within the same environment should be used to compose the batch and expand its genetic base.

### Haplotype diversity based on cpSSR

When assessing genetic diversity through the lens of maternal inheritance (cpSSR markers), *P. reticulata* populations showed a low *N*_*H*_. This finding aligns with the pattern of low to moderate diversity observed for biparental inheritance (nSSR markers). We believe that the low diversity of haplotypes results from the origin and distribution pattern of the species. A phylogeographic study suggested that *P. reticulata* originates from the Savanna of central Brazil, which also corresponds to its centre of genetic diversity (Novaes *et al.* 2010). In this context, we assumed that the species had recently radiated from this central region to Brazil’s Northeast region (location of the populations under study), which could explain the high rate (72.3 %) of individuals presenting the same haplotype (Novaes *et al.* 2010).

We believe that the higher values in genetic variability descriptors generally reported for Savanna compared to Forest are due to the environmental similarity with the centre of origin and diversity of *P. reticulata*, which is a Savanna species. Therefore, we would expect greater colonization success for migrants of the species moving between similar habitats, that is, from Savanna to Savanna than between dissimilar habitats, such as Savanna and Forest. This could potentially lead to a higher diversity of haplotypes in Savanna environment. However, this hypothesis needs to be tested through field translocation experiments and by using a larger number of chloroplast markers to obtain more conclusive results.

### Genetic status and implications for the genus Plathymenia

*Plathymenia reticulata* is a species whose taxonomic classification has undergone several revisions since its initial description. These reviews present divergent results, suggesting that the genus Plathymenia could be composed of a single species (Ducke 1925; Warwick and Lewis 2003), two species (Bentham 1842; Heringer 1956) or two species and a variant (Burkart 1939). In the present study, we assume that it represents a unique species (Warwick and Lewis 2003), found in ecotypes in the Atlantic Forest and Cerrado (Lemos *et al.* 2008).

Based on this classification, we demonstrated that in addition to the phenotypic differentiation reported in studies comparing seed and fruit morphology (Goulart *et al.* 2006; Lopes *et al.* 2010) and leaf attributes (Mendes and Paviani 1997), *P. reticulata* shows genetic differentiation among populations occurring in different biomes. A similar pattern was also observed in populations from the Savanna and Atlantic Forest in the state of Minas Gerais, southeastern Brazil (Muniz *et al.* 2022, 2023). This suggests the need for a taxonomic review of the genus, aimed at confirming the potential existence of two species within the genus Plathymenia, first described by Bentham in 1842. From this perspective, distinguishing *P. foliolosa* from *P. reticulata*, or dividing *P. reticulata* into two ecotypes to identify significant evolutionary units, could enhance management and conservation strategies for the genus. This includes addressing their vulnerability status on the IUCN list (IUCN 2021) and developing seed translocation strategies for ecosystem restoration. Thus, although these evolutionary units are not reproductively isolated (Muniz *et al.* 2022), we demonstrated significant genetic differentiation between the Savanna and Forest populations in the state of Bahia, northeast Brazil.

Furthermore, we characterized native populations as seed sources and emphasized the importance of conserving their unique characteristics, given their potential as phylogenetic resources. Thus, preserving populations as significant evolutionary units with unique compositions and allelic frequencies is essential. This approach ensures the continuation of the species’ evolutionary path, including the accumulation of both naturally occurring and selectively advantageous alleles for each adaptive environment.

## Conclusions

We demonstrated through the genetic characterization of nine native populations of *Plathymenia reticulata*, located in two Brazilian biodiversity hotspots, low to moderate levels of variability and a strong global genetic structure. We emphasize the differences in the gene pool composition between the Savanna and Atlantic Forest populations, with soil variables, climatic conditions and spatial distance contributing significantly to this differentiation. Thus, we present new scientific findings for the northeast region of Brazil, which align with morphological and genetic studies of this tree species in the southeast region of the country. These studies have demonstrated that phytophysiognomy serves as a criterion for distinguishing two significant evolutionary units. Given the significant genetic differences between Savanna and Forest populations, the translocation of *P. reticulata* seeds should be carried out according to biome, with the purchase or collection of seeds limited to the environment in which they will be planted. Furthermore, due to the reported low or moderate genetic diversity, we recommend that batches of seeds for restoration purposes should include different populations from within each biome. This approach aims to expand the genetic base of the populations to be established. We also emphasize the importance of preserving the remaining populations as a gene bank for future restoration projects and long-term management strategies. This aims to establish populations that are adapted to environmental conditions and more resilient to climate change. Finally, we emphasize that during the decade of ecosystem restoration, the restoration process should not be viewed in isolation. Strategies ensuring the conservation of existing populations, including from a genetic perspective, are crucial.

## Supporting Information

The following additional information is available in the online version of this article –

**Table S1.** Analysis of molecular variance (AMOVA) for nine populations of *P. reticulata*, inserted in two phytophysiognomy (Forest and Savanna) and four climates (Af, Aw, Cfb and Bsh) in the state of Bahia, Brazil.

**Table S2.** Simple Mantel test for genetic, environment (edaphic and bioclimatic), and spatial distance for nine populations of *P. reticulata* in Bahia, Brazil.

**Table S3.** Variables identified in distance-based redundancy analysis (*dbRDA*) as significantly associated with *P. reticulata* gene pool distribution in the populations evaluated in the study.

**Table S4** **.** Proportion of the genetic structure of the nine *P. reticula* populations sampled in Bahia, Brazil, explained by the *RDA1* and *RDA2* axes and the relationship of *BIO2* and *MEM1* with the *RDA1* and *RDA2* axes, identified in distance-based redundancy analysis (*dbRDA*).

**Table S5.** Haplotype frequency observed in *P. reticulata* according to populations, Forest and Savanna, and in climatic groups in Bahia, Brazil. CAM_NF = Camacan, IBI_NF = Ibirapitanga, JUS_NF = Jussarí, UNA_NF = UNA, AMA_NF = Amargosa, ABA_NS = Abaíra, LEN_NS = Lençóis, MIG_NS = Miguel Calmon and MUC_NS = Mucugê. NF= Native Forest and NS= Native Savanna.

**Table S6.** Haplotypic diversity parameters for the *P. reticulata* populations studied based on three chloroplast simple sequence repeats (cpSSR): *N* = number of individuals, *N_H_* = number of haplotypes, *H_Ne_* = effective number of haplotypes, *PH* = private haplotypes, *H* = Haplotypic richness and *H_E_* = haplotypic diversity and *D^2^sh* = distance between individuals. CAM_NF = Camacan, IBI_NF = Ibirapitanga, JUS_NF = Jussarí, UNA_NF = UNA, AMA_NF = Amargosa, ABA_NS = Abaíra, LEN_NS = Lençóis, MIG_NS = Miguel Calmon and MUC_NS = Mucugê. NF = Native Forest and NS = Native Savanna.

**Table S7.** Haplotypic diversity parameters for the *P. reticulata* populations in different Vegetation and Climatic typology studied based on three chloroplast simple sequence repeats (cpSSR): *N* = number of individuals, *N_H_* = number of haplotypes, *H_Ne_* = effective number of haplotypes, *P_H_* = private haplotypes , *H* = Haplotypic richness, *H_E_* = haplotypic diversity and *D^2^sh* = distance between individuals.

**Table S8.** Normalized Pairwise *F_ST_* with cpSSR for nine populations of *P. reticulata* in Bahia, Brazil. CAM_NF = Camacan, IBI_NF = Ibirapitanga, JUS_NF = Jussarí, UNA_NF = UNA, AMA_NF = Amargosa, ABA_NS = Abaíra, LEN_NS = Lençóis, MIG_NS = Miguel Calmon and MUC_NS = Mucugê. NF = Native Forest and NS = Native Savanna.

**Figure S1.** Different structures of *Plathymenia reticulata*. A: Trunk; B: Leaves; C: inflorescence; D: Fruits and seeds surrounded by membrane and seed with radicle protrusion. The fruits measure approximately 7.8–16 × 1.4–3.6 cm, the seeds 6–14 × 4–8 mm.

**Figure S2.** Map of South America, highlighting Brazil, Bahia and their climate types. (A) Koppen climate classification for Brazil; (B) geographic location of sampled populations of *Plathymenia reticulata* in climate types Af, Aw, Cfb and Bsh in Bahia, according to Koppen’s classification for Brazil (Alvares *et al.* 2013); (C) sampling scheme with the climatic types studied Af (dark blue), Aw (beige), Cfb (black) and Bsh (yellow) with the populations that compose them. CAM_NF = Camacan, IBI_NF = Ibirapitanga, JUS_NF = Jussarí, UNA_NF = UNA, AMA_NF = Amargosa, ABA_NS = Abaíra, LEN_NS = Lençóis, MIG_NS = Miguel Calmon and MUC_NS = Mucugê. NF = Native Forest and NS = Native Savanna.

**Figure S3.** Correlation between edaphic variables. Values of variation inflation factor (*VIF*) for soil variables. *Cec* = soil cation exchange capacity; *phh2o* = soil pH; *soc* = soil organic carbon content. *Cec*, *phh2o* and *soc* present *VIF* values ≤ 5.

**Figure S4.** Correlation between bioclimatic variables Values of variation inflation factor (*VIF*) for clim variables. *BIO2* =Mean Diurnal Range; *BIO12* =Annual Precipitation. *BIO2* and *BIO12* present *VIF* values ≤ 5.

**Figure S5.** Genetic spatial principal component analysis (*sPCA*) for nine populations of *P. reticulata* in Bahia, Brazil, with the scores for each *sPCA* axis explaining local (blue) and global (red) genetic structure. As the first two axes of the *sPCA* presented the highest scores, they were used as a measure of the genetic structure in distance-based redundancy analysis (*dbRDA*).

**Figure S6.** Pairwise *F_ST_* estimated with nuclear microsatellite markers (nSSR) for nine populations of *P. reticulata* in Bahia, Brazil. Lighter blues show lower *F_ST_* values, and darker tones show higher *F_ST_* values. CAM_NF, IBI_NF, JUS_NF, UNA_NF and AMA_NF represent populations of Forest. ABA_NS, LEN_NS, MIG_NS and MUC_NS represent populations of Savanna.

**Figure S7.** Spatial genetic structure with nuclear microsatellite markers (nSSR) for nine populations of *P. reticulata* in Bahia, Brazil, based on the first two scores of the genetic spatial principal component analysis (*sPCA*). Evidencing K = 2, with a group composed of populations located in Savanna and another group formed by populations located in Forest (composed of two subgroups, highlighted in green and blue). CAM_NF = Camacan, IBI_NF = Ibirapitanga, JUS_NF = Jussarí, UNA_NF = UNA, AMA_NF = Amargosa, ABA_NS = Abaíra, LEN_NS = Lençóis, MIG_NS = Miguel Calmon and MUC_NS = Mucugê. NF = Native Forest and NS = Native Savanna.

**Figure S8.** Venn diagram with the proportion of variance in genetic structure for nine populations of *P. reticulata*, explained by spatial variation (spatial predictor, *MEM1*) and *BIO2* = Average Diurnal Interval (maximum temperature - minimum temperature).

**Figure S9.** Simple Mantel test used to explore the relationship between genetic distance (*F_ST_*) and geographic distance (Km) between pairs of nine populations of *P. reticulata* in Bahia, Brazil, by chloroplast microsatellite markers (cpSSR). (r = -0.06, P = 0.542).

plae032_suppl_Supplementary_Material

plae032_suppl_Supplementary_Data

## Data Availability

All raw data used to design the manuscript are available in the supplementary material (‘Genotyping data.xlsx’).

## References

[CIT0001] Aavik T , Helm, A .2018. Restoration of plant species and genetic diversity depends on landscape-scale dispersal. Restoration Ecology 26:92–102.

[CIT0002] Almeida SP , Centro de Pesquisa Agropecuária dos Cerrados. 1998. Cerrado: Espécies Vegetais Úteis. Planaltina, DF: Embrapa - CPAC.

[CIT0003] Alvares CA , Stape JL, Sentelhas PC, de Moraes Gonçalves JL, Sparovek G. 2013. Köppen’s climate classification map for Brazil. Meteorologische Zeitschrift 22:711–728.

[CIT0004] Araújo FM , Ferreira LG, Arantes AE. 2012. Distribution patterns of burned areas in the Brazilian biomes: an analysis based on satellite data for the 2002–2010 period. Remote Sensing 4:1929–1946.

[CIT0005] Aronson J , Goodwin N, Orlando L, Eisenberg C, Cross AT. 2020. A world of possibilities: six restoration strategies to support the United Nation’s Decade on Ecosystem Restoration. Restoration Ecology 28:730–736.

[CIT0006] Axelsson EP , Grady KC, Lardizabal ML, Nair IB, Rinus D, Ilstedt U. 2020. A pre-adaptive approach for tropical Forest restoration during climate change using naturally occurring genetic variation in response to water limitation. Restoration Ecology 28:156–165.

[CIT0008] Basey AC , Fant JB, Kramer AT. 2015. Producing native plant materials for restoration: 10 rules to collect and maintain genetic diversity. Native Plants Journal 16:37–53.

[CIT0009] Bentham G. 1842. Mimoseae. Hook. Journal of Botany 4:333–334.

[CIT0010] Betts MG , Wolf C, Pfeifer M, Banks-Leite C, Arroyo-Rodríguez V, Ribeiro DB, Barlow J, Eigenbrod F, Faria D, Fletcher RJ, et al. 2019. Extinction filters mediate the global effects of habitat fragmentation on animals. Science 366:1236–1239. 31806811 10.1126/science.aax9387

[CIT0011] Boshier D , Broadhurst L, Cornelius J, Gallo L, Koskela J, Loo J, Petrokofsky G, St Clair B. 2015. Is local best? Examining the evidence for local adaptation in trees and its scale. Environmental Evidence 4:1–10.

[CIT0012] Bradburd GS , Ralph PL, Coop GM. 2013. Disentangling the effects of geographic and ecological isolation on genetic differentiation. Evolution 67:3258–3273. 24102455 10.1111/evo.12193 PMC3808528

[CIT0013] Breed MF , Harrison PA, Bischoff A, Durruty P, Gellie NJC, Gonzales EK, Havens K, Karmann M, Kilkenny FF, Krauss SL, et al. 2018. Priority actions to improve provenance decision-making. BioScience 68:510–516.

[CIT0015] Broadhurst L , Van Rossum F, Jones T, Jordan R, Encinas-Viso F, Harrison PA, Broadhurst L, Encinas-Viso F. 2023. Restoration genetics—a consideration of lessons and opportunities. In Florentine S , Gibson-Roy P, Dixon KW, Broadhurst L, eds. Ecological restoration: moving forward using lessons learned. Cham: Springer International Publishing, 473–519.

[CIT0016] Bucharova A , Bossdorf O, Hölzel N, Kollmann J, Prasse R, Durka W. 2019. Mix and match: regional admixture provenancing strikes a balance among different seed-sourcing strategies for ecological restoration. Conservation Genetics 20:7–17.

[CIT0007] Burkart, A. 1939. Leguminosas: mimosoideas. Itajaí: Herbário Barbosa Rodrigues, 299.

[CIT0017] Carvalho PER. 2009. Vinhático—*Plathymenia reticulata*. *Embrapa Florestas. Comunicado Técnico* 231:1517–5030.

[CIT0018] Colli GR , Vieira CR, Dianese JC. 2020. Biodiversity and conservation of the Cerrado: recent advances and old challenges. Biodiversity and Conservation 29:1465–1475.

[CIT0019] Cordeiro EMG , Macrini CM, Sujii PS, Schwarcz KD, Pinheiro JB, Rodrigues RR, Brancalion PHS, Zucchi MI. 2019. Diversity, genetic structure, and population genomics of the tropical tree *Centrolobium tomentosum* in remnant and restored Atlantic forests. Conservation Genetics 20:1073–1085.

[CIT0020] Coutinho, L. 2016. Brazilian biomes. Text Workshop.

[CIT0126] de Souza EMS , Álvares-Carvalho SV, Ferreira RA, Silva-Mann R. 2022. *Schinus terebinthifolia* Raddi: a comparative framework on population genetic structure in a restored area after 12 years. *Genetic Resources and Crop Evolution* 69:2459–2467.

[CIT0021] Doyle JJ , Doyle JL. 1987. A rapid DNA isolation procedure for small quantities of fresh leaf tissue. Phytochemical Bulletin 19:11–15.

[CIT0024] Dray S , Pélissier R, Couteron P, Fortin M-J, Legendre P, Peres-Neto PR, Bellier E, Bivand R, Blanchet FG, De Cáceres M, et al. 2012. Community ecology in the age of multivariate multiscale spatial analysis. Ecological Monographs 82:257–275.

[CIT0022] Ducke WA. 1925. As leguminosas do Estado do Para. Archivos do Jardim Botânico do Rio de Janeiro 4:253.

[CIT0023] Dupré La Tour A , Labatut J, Spiegelberger T. 2020. Unraveling the concept of local seeds in restoration ecology. Restoration Ecology 28:1327–1334.

[CIT0025] Earl DA , Vonholdt BM. 2012. STRUCTURE HARVESTER: a website and program for visualizing STRUCTURE output and implementing the Evanno method. Conservation Genetics Resources 4:359–361.

[CIT0026] Edmands S. 2007. Between a rock and a hard place: evaluating the relative risks of inbreeding and outbreeding for conservation and management. Molecular Ecology 16:463–475. 17257106 10.1111/j.1365-294X.2006.03148.x

[CIT0027] Edwards DP , Socolar JB, Mills SC, Burivalova Z, Koh LP, Wilcove DS. 2019. Conservation of tropical forests in the anthropocene. Current Biology 29:R1008–R1020. 31593660 10.1016/j.cub.2019.08.026

[CIT0028] Eliades NG , Eliades DG. 2009. User’s manual. Software for Analysis of Haplotype, Haplotype Analysis Data.

[CIT0030] Erickson VJ , Halford A. 2020. Seed planning, sourcing, and procurement. Restoration Ecology 28:S216–S224.

[CIT0031] Excoffier L , Lischer HEL. 2010. Arlequin suite ver 3.5: a new series of programs to perform population genetics analyses under Linux and Windows. Molecular Ecology Resources 10:564–567. 21565059 10.1111/j.1755-0998.2010.02847.x

[CIT0032] Faria D , Morante-Filho JC, Baumgarten J, Bovendorp RS, Cazetta E, Gaiotto FA, Mariano-Neto E, Mielke MS, Pessoa MS, Rocha-Santos L, et al. 2023. The breakdown of ecosystem functionality driven by deforestation in a global biodiversity hotspot. Biological Conservation 283:110126.

[CIT0033] Fernandes AKC , Quadros TMC, Conceição TA, Waqar Z, Cardoso I, Santos AS, Gaiotto FA. 2023. Can forest restoration affect the genetic diversity of plants? Ecological Restoration 41:152–157.

[CIT0034] Forrest NC , Ottewell KM, Whelan RJ, Ayre DJ. 2011. Tests for inbreeding and outbreeding depression and estimation of population differentiation in the bird-pollinated shrub *Grevillea mucronulata*. Annals of Botany 108:185–195. 21546431 10.1093/aob/mcr100 PMC3119612

[CIT0036] Frankham R , Ballou JD, Eldridge MD, Lacy RC, Ralls K, Dudash MR, Fenster CB. 2011. Predicting the probability of outbreeding depression. *Conservation Biology* 25:465–475. 21486369 10.1111/j.1523-1739.2011.01662.x

[CIT0035] Funch RR , Harley RM, Funch LS. 2009. Mapping and evaluation of the state of conservation of the vegetation in and surrounding the Chapada Diamantina National Park, NE Brazil. Biota Neotropica 9:21–30.

[CIT0037] Gann GD , McDonald T, Walder B, Aronson J, Nelson CR, Jonson J, Hallett JG, Eisenberg C, Guariguata MR, Liu J, et al. 2019. International principles and standards for the practice of ecological restoration. Second edition. Restoration Ecology 27:S1–S46.

[CIT0038] GBIF Secretariat. 2023. *Plathymenia reticulata* Benth in GBIF backbone taxonomy. Checklist dataset. doi:10.15468/39omei. Accessed 17 March 2023.

[CIT0039] Giencke LM , Carol Denhof R, Katherine Kirkman L, Stribling Stuber O, Brantley ST. 2018. Seed sourcing for longleaf pine ground cover restoration: using plant performance to assess seed transfer zones and home-site advantage. *Restoration Ecology* 26:1127–1136.

[CIT0042] Goto S , Iijima H, Ogawa H, Ohya K. 2011. Outbreeding depression caused by intraspecific hybridization between local and nonlocal genotypes in *Abies sachalinensis*. Restoration Ecology 19:243–250.

[CIT0040] Goudet J. 1995. FSTAT (version 1.2): a computer program to calculate *F*-statistics. Journal of Heredity 86:485–486.

[CIT0041] Goulart MF , Pires Lemos Filho J, Lovato MB. 2006. Variability in fruit and seed morphology among and within populations of *Plathymenia* (Leguminosae—Mimosoideae) in areas of the Cerrado, the Atlantic Forest, and transitional sites. Plant Biology 8:112–119. 16435275 10.1055/s-2005-865964

[CIT0043] Griscom BW , Adams J, Ellis PW, Houghton RA, Lomax G, Miteva DA, Schlesinger WH, Shoch D, Siikamäki JV, Smith P, et al. 2017. Natural climate solutions. Proceedings of the National Academy of Sciences of the United States of America 114:11645–11650. 29078344 10.1073/pnas.1710465114 PMC5676916

[CIT0044] Hancock N , Hughes L. 2014. Aumentando o debate sobre a proveniência: testar o paradigma ‘local é o melhor’ sob condições de ondas de calor. Ecologia Austral 39:600–611.

[CIT0045] Hansen MC , Potapov PV, Moore R, Hancher M, Turubanova SA, Tyukavina A, Thau D, Stehman SV, Goetz SJ, Loveland TR, et al. 2013. High-resolution global maps of 21st-century forest cover change. Science 342:850–853. 24233722 10.1126/science.1244693

[CIT0046] Harley, RM. 1995. Introduction. In: BL. Stannard, ed. Flora of the Pico das Almas, Chapada Diamantina, Brazil. Kew: Royal Botanic Gardens, 853

[CIT0047] Heringer EP. 1956. O gênero *Plathymenia*. Anais da Sociedade Botânica do Brasil 7:55–64.

[CIT0048] Hoban S , Schlarbaum S. 2014. Optimal sampling of seeds from plant populations for ex-situ conservation of genetic biodiversity, considering realistic population structure. Biological Conservation 177:90–99.

[CIT0049] Hoban S , Strand A. 2015. Ex situ seed collections will benefit from considering spatial sampling design and species’ reproductive biology. Biological Conservation 187:182–191.

[CIT0050] Hufford KM , Krauss SL, Veneklaas EJ. 2012. Inbreeding and outbreeding depression in *Stylidium hispidum*: implications for mixing seed sources for ecological restoration. Ecology and Evolution 2:2262–2273. 23139884 10.1002/ece3.302 PMC3488676

[CIT0051] Isbell F , Gonzalez A, Loreau M, Cowles J, Díaz S, Hector A, Mace GM, Wardle DA, O'Connor MI, Duffy JE, et al. 2017. Linking the influence and dependence of people on biodiversity across scales. Nature 546:65–72. 28569811 10.1038/nature22899 PMC5460751

[CIT0052] IUCN. 2021. The IUCN red list of threatened species. Version 2021-3. https://www.iucnredlist.org. Accessed 26 April 2022.

[CIT0053] Jalonen R , Valette M, Boshier D, Duminil J, Thomas E. 2018. Forest and landscape restoration severely constrained by a lack of attention to the quantity and quality of tree seed: insights from a global survey. Conservation Letters 11:e12424.

[CIT0054] Jia Y , Milne RI, Zhu J, Gao L-M, Zhu G-F, Zhao G-F, Liu J, Li Z-H. 2020. Evolutionary legacy of a forest plantation tree species (*Pinus armandii*): implications for widespread afforestation. Evolutionary Applications 13:2646–2662. 33294014 10.1111/eva.13064 PMC7691453

[CIT0055] Jombart TA. 2008. A R package for the multivariate analysis of genetic markers. Bioinformatics 24:1403–1405. 18397895 10.1093/bioinformatics/btn129

[CIT0056] Jombart T , Ahmed I. 2011. Adegenet 1.3-1: new tools for the analysis of genome-wide SNP data. Bioinformatics 27:3070–3071. 21926124 10.1093/bioinformatics/btr521 PMC3198581

[CIT0057] Jorgensen MH , Elameen A, Hofman N, Klemsdal S, Malaval S, Fjellheim S. 2016. What’s the meaning of local? Using molecular markers to define seed transfer zones for ecological restoration in Norway. Evolutionary Applications 9:673–684. 27247618 10.1111/eva.12378 PMC4869409

[CIT0058] Kashimshetty Y , Pelikan S, Rogstad SH. 2017. Effective seed harvesting strategies for the ex situ genetic diversity conservation of rare tropical tree populations. Biodiversity and Conservation 26:1311–1331.

[CIT0060] Keller M , Kollmann J, Edwards PJ. 2000. Genetic introgression from distant provenances reduces fitness in local weed populations. Journal of Applied Ecology 37:647–659.

[CIT0061] Kettenring KM , Tarsa EE. 2020. Need to seed? Ecological, genetic, and evolutionary keys to seed-based wetland restoration. Frontiers in Environmental Science 8:109.

[CIT0063] Krauss SL , Sinclair EA, Bussell JD, Hobbs RJ. 2013. An ecological genetic delineation of local seed-source provenance for ecological restoration. Ecology and Evolution 3:2138–2149. 23919158 10.1002/ece3.595 PMC3728953

[CIT0064] Kucera KF , Fant JB, Jensen S, Landeen M, Orr E, Kramer AT. 2022. Genetic variation and structure change when producing and using mixed-source seed lots for restoration. Restoration Ecology 30:e13521.

[CIT0066] Legendre P , Legendre L. 2014. Numerical ecology. Developments in environmental modelling, 3rd ed., vol. 24. Elsevier.

[CIT0067] Lemos Filho JP , Goulart MF, Lovato MB. 2008. Populational approach in ecophysiological studies: the case of *Plathymenia reticulata*, a tree from Cerrado and Atlantic Forest. Brazilian Journal of Plant Physiology 20:205–216.

[CIT0068] Liddell E , Sunnucks P, Cook CN. 2021. To mix or not to mix gene pools for threatened species management? Few studies use genetic data to examine the risks of both actions, but failing to do so leads disproportionately to recommendations for separate management. Biological Conservation 256:109072.

[CIT0070] Lima RA , Dauby G, de Gasper AL, Fernandez EP, Vibrans AC, Oliveira AA, Prado PI, Souza VC, F. de Siqueira M, Ter Steege H. 2024. Comprehensive conservation assessments reveal high extinction risks across Atlantic Forest trees. Science 383:219–225. 38207046 10.1126/science.abq5099

[CIT0069] Lima RA , Oliveira AA, Pitta GR, de Gasper AL, Vibrans AC, Chave J, Ter Steege H, Prado PI. 2020. The erosion of biodiversity and biomass in the Atlantic Forest biodiversity hotspot. Nature Communications 11:6347 10.1038/s41467-020-20217-w PMC7733445 33311511

[CIT0071] Lopes RMF , Freitas VLO, Lemos-Filho JP. 2010. Biometria de frutos e sementes e germinação de *Plathymenia reticulata* Benth. e *Plathymenia foliolosa* benth. (Fabaceae—mimosoideae). Revista Árvore 34:797–805.

[CIT0072] Maracahipes L , Carlucci MB, Lenza E, Marimon BS, Marimon BH, Guimarães FAG, Cianciaruso MV. 2018. How to live in contrasting habitats? Acquisitive and conservative strategies emerge at inter- and intraspecific levels in Savanna and Forest woody plants. Perspectives in Plant Ecology, Evolution and Systematics 34:17–25.

[CIT0073] Mckay JK , Christian CE, Harrison S, Rice KJ. 2005. ‘How Local Is Local?’ A review of practical and conceptual issues in the genetics of restoration. Restoration Ecology 13:432–440.

[CIT0075] Meissen JC , Galatowitsch SM, Cornett MW. 2015. Risks of overharvesting seed from native tallgrass prairies. Restoration Ecology 23:882–891.

[CIT0076] Meissen JC , Galatowitsch SM, Cornett MW. 2017. Meeting seed demand for landscape-scale restoration sustainably: the influence of seed harvest intensity and site management. Écoscience 24:1–11.

[CIT0074] Mendes IDCA , Paviani TI. 1997. Morfo-anatomia comparada das folhas do par vicariante *Plathymenia foliolosa* Benth. e *Plathymenia reticulata* Benth. (Leguminosae - Mimosoideae). Revista Brasileira de Botânica 20:185–195.

[CIT0077] Mijangos JL , Pacioni C, Spencer PBS, Craig MD. 2015. Contribution of genetics to ecological restoration. Molecular Ecology 24:22–37. 25377524 10.1111/mec.12995

[CIT0078] Mitchard ETA. 2018. The tropical Forest carbon cycle and climate change.*Nature* 559:527–534. 30046067 10.1038/s41586-018-0300-2

[CIT0079] Moreno-Mateos D , Alberdi A, Morriën E, van der Putten WH, Rodríguez-Uña A, Montoya D. 2020. The long-term restoration of ecosystem complexity. Nature Ecology & Evolution 4:676–685. 32284582 10.1038/s41559-020-1154-1

[CIT0080] Muniz AC , Pimenta RJG, Cruz MV, Rodrigues JG, Buzatti RSO, Heuertz M, Lemos-Filho JP, Lovato MB. 2022. Hybrid zone of a tree in a Cerrado/Atlantic Forest ecotone as a hotspot of genetic diversity and conservation. Ecology and Evolution 12:e8540. 35127043 10.1002/ece3.8540 PMC8803295

[CIT0081] Muniz AC , de Oliveira Buzatti RS, de Lemos-Filho JP, Heuertz M, Nazareno AG, Lovato MB. 2023. Genomic signatures of ecological divergence between savanna and forest populations of a Neotropical tree. Annals of Botany 132:523–540. 37642427 10.1093/aob/mcad120 PMC10667007

[CIT0082] Myers N , Mittermeier RA, Mittermeier CG, da Fonseca GA, Kent J. 2000. Biodiversity hotspots for conservation priorities. Nature 403:853–858. 10706275 10.1038/35002501

[CIT0083] Nagamitsu T , Shuri K. 2021. Seed transfer across geographic regions in different climates leads to reduced tree growth and genetic admixture in *Quercus mongolica* var. crispula. Forest Ecology and Management 482:118787.

[CIT0084] Neves SP , Funch R, Conceição AA, Miranda LA, Funch LS. 2016. What are the most important factors determining different vegetation types in the Chapada Diamantina, Brazil? Brazilian Journal of Biology 76:315–333. 10.1590/1519-6984.13814 26934155

[CIT0085] Nevill PG , Tomlinson S, Elliott CP, Espeland EK, Dixon KW, Merritt DJ. 2016. Seed production areas for the global restoration challenge. Ecology and Evolution 6:7490–7497. 28725415 10.1002/ece3.2455 PMC5513262

[CIT0086] Novaes RM , De Lemos Filho JP, Ribeiro RA, Lovato MB. 2010. Phylogeography of *Plathymenia reticulata* (Leguminosae) reveals patterns of recent range expansion towards northeastern Brazil and southern Cerrados in Eastern Tropical South America. Molecular Ecology 19:985–998. 20149092 10.1111/j.1365-294X.2010.04530.x

[CIT0087] Oksanen J , Simpson GL, Blanchet FG, Kindt R, Legendre P, Minchin PR, O’Hara RB, Solymos P, Stevens MHH, Szoecs E, et al. 2020. vegan: community ecology package. Community ecology package, version, 2, 9: 1-295, R package version 2.5- 7. https://CRAN.R-project.org/package=vega

[CIT0088] Oliveira, FA. 2012. Estrutura genética espacial e conservação de *Plathymenia reticulata* na paisagem agroflorestal cacaueira-‘cabrucas’. Ilhéus: Universidade Estadual de Santa Cruz, Programa De Pós-Graduação em Genética e Biologia Molecular.

[CIT0089] Oliveira FA , Tarazi R, Menezes IPP, Van Den Berg C, Tsai SM, Gaiotto FA. 2012. Microsatellite markers for *Plathymenia reticulata* (Leguminosae). American Journal of Botany 99:e391–e393. 22986084 10.3732/ajb.1200051

[CIT0090] Peakall R , Smouse PE. 2006. GENALEX 6: genetic analysis in Excel. Population genetic software for teaching and research. Molecular Ecology Notes 6:288–295. 10.1093/bioinformatics/bts460 PMC3463245 22820204

[CIT0091] Poggio L , de Sousa LM, Batjes NH, Heuvelink GBM, Kempen B, Ribeiro E, Rossiter D. 2021. SoilGrids 2.0: producing soil information for the globe with quantified spatial uncertainty. Soil 7:217–240.

[CIT0093] Pritchard JK , Stephens M, Donnelly P. 2000. Inference of population structure using multilocus genotype data. Genetics 155:945–959. 10835412 10.1093/genetics/155.2.945 PMC1461096

[CIT0094] Proft KM , Jones ME, Johnson CN, Burridge CP. 2018. Making the connection: expanding the role of restoration genetics in restoring and evaluating connectivity. Restoration Ecology 26:411–418.

[CIT0095] Ribeiro JF , Kuhlmann M, Santos DS, Sampaio A, Ogata RS, Souza RM, Oliveira MC, Durigan G, Munhoz CBR, Valls JFM, et al. 2018. Espécies vegetais nativas recomendadas para recomposição ambiental no bioma Cerrado. Embrapa Cerrados Planaltina, DF.

[CIT0097] Rutherford S , van der Merwe M, Wilson PG, Kooyman RM, Rossetto M. 2019. Managing the risk of genetic swamping of a rare and restricted tree. Conservation Genetics 20:1113–1131.

[CIT0099] Sambuichi RHR , Vidal DB, Piasentin FB, Jardim JG, Viana TG, Menezes AA, Mello DLN, Ahnert D, Baligar VC. 2012. Cabruca agroForests in southern Bahia, Brazil: tree component, management practices and tree species conservation. Biodiversity and Conservation 21:1055–1077.

[CIT0100] Santana NS , Santos AS, Borges DB, de Souza França D, Reis JB, de Oliveira FA, Barreto MA, Corrêa RX, Zucchi MI, Martins K, et al. 2023. Genetic resilience of Atlantic forest trees to impacts of biome loss and fragmentation. European Journal of Forest Research 142:161–174.

[CIT0102] Santos GL , Pereira MG, Delgado RC, Magistrali IC, Silva CG, Oliveira CM, Larangeira JP, Silva TP. 2021. Degradation of the Brazilian Cerrado: interactions with human disturbance and environmental variables. Forest Ecology and Management 482:118875.

[CIT0101] Santos AS , Cazetta E, Faria D, Lima TM, Lopes MTG, Carvalho CS, Alves-Pereira A, Morante-Filho JC, Gaiotto FA. 2023. Tropical forest loss and geographic location drive the functional genomic diversity of an endangered palm tree. Evolutionary Applications 16:1257–1273. 37492151 10.1111/eva.13525 PMC10363835

[CIT0103] Schäfer D , Vincent H, Fischer M, Kempel A. 2020. The importance of genetic diversity for the translocation of eight threatened plant species into the wild. Global Ecology and Conservation 24:e01240.

[CIT0104] Sexton JP , Hangartner SB, Hoffmann AA. 2014. Genetic isolation by environment or distance: which pattern of gene flow is most common? Evolution 68:1–15. 24111567 10.1111/evo.12258

[CIT0105] Shaw J , Lickey EB, Schilling EE, Small RL. 2007. Comparison of whole chloroplast genome sequences to choose noncoding regions for phylogenetic studies in angiosperms: the tortoise and the hare III. American Journal of Botany 94:275–288. 21636401 10.3732/ajb.94.3.275

[CIT0106] SOS Mata Atlântica; INPE. 2022. Atlas dos remanescentes florestais da Mata Atlântica: período 2020-2021. SOS Mata Atlântica and INPE, SP.

[CIT0108] Stange M , Barrett RDH, Hendry AP. 2021. The importance of genomic variation for biodiversity, ecosystems and people. Nature Reviews Genetics 22:89–105. 10.1038/s41576-020-00288-7 33067582

[CIT0109] Sujii PS , Schwarcz KD, Grando C, de Aguiar Silvestre E, Mori GM, Brancalion PHS, Zucchi MI. 2017. Recovery of genetic diversity levels of a Neotropical tree in Atlantic Forest restoration plantations. Biological Conservation 211:110–116.

[CIT0110] Sujii PS , Tambarussi EV, Grando C, de Aguiar Silvestre E, Viana JPG, Brancalion PHS, Zucchi MI. 2021. High gene flow through pollen partially compensates spatial limited gene flow by seeds for a Neotropical tree in forest conservation and restoration areas. Conservation Genetics 22:383–396.

[CIT0111] Thomas E , Jalonen R, Loo J, Boshier D, Gallo L, Cavers S, Bordács S, Smith P, Bozzano M. 2014. Genetic considerations in ecosystem restoration using native tree species. Forest Ecology and Management 333:66–75.

[CIT0112] Van Der Merwe MM , Yap JY, Wilson PD, Murphy HT, Ford A. 2021. All populations matter: conservation genomics of australia’s iconic purple wattle, *Acacia purpureopetala*. Diversity 13:139.

[CIT0113] Van Oosterhout C , Hutchinson WF, Wills D, Shipley P. 2004. MICRO-CHECKER: software for identifying and correcting genotyping errors in microsatellite data. Molecular Ecology Notes 4:535–538.

[CIT0114] Van Rossum F , Le Pajolec S, Raspé O, Godé C. 2022. Assessing population genetic status for designing plant translocations. Frontiers in Conservation Science 3:829332.

[CIT0115] Warwick MC , Lewis, GP. 2003. Revision of *Plathymenia* (Leguminosae–Mimosoideae). Edinburgh Journal of Botany 60:111–119.

[CIT0116] Weeks AR , Sgro CM, Young AG, Frankham R, Mitchell NJ, Miller KA, Byrne M, Coates DJ, Eldridge MDB, Sunnucks P, et al. 2011. Assessing the benefits and risks of translocations in changing environments: a genetic perspective. Evolutionary Applications 4:709–725. 22287981 10.1111/j.1752-4571.2011.00192.x PMC3265713

[CIT0117] Wei X , Xu Y, Lyu L, Xiao Z, Wang S, Yang T, Jiang M. 2023. Impacts of ecological restoration on the genetic diversity of plant species: a global meta‐analysis. Journal of Applied Ecology 60:1149–1160.

[CIT0118] Weidlich EW , Nelson CR, Maron JL, Callaway RM, Delory BM, Temperton VM. 2021. Priority effects and ecological restoration. Restoration Ecology 29:e13317.

[CIT0119] Weising K , Gardner RC. 1999. A set of conserved PCR primers for the analysis of simple sequence repeat polymorphisms in chloroplast genomes of dicotyledonous angiosperms. Genome 42:9–19. 10207998

[CIT0120] Whitlock MC. 2008. Evolutionary inference from QST. Molecular Ecology 17:1885–1896. 18363667 10.1111/j.1365-294X.2008.03712.x

[CIT0121] Wicke S , Schneeweiss GM, dePamphilis CW, Müller KF, Quandt D. 2011. The evolution of the plastid chromosome in land plants: gene content, gene order, gene function. Plant Molecular Biology 76:273–297. 21424877 10.1007/s11103-011-9762-4 PMC3104136

[CIT0122] Woolridge CB , Fant JB, Flores AI, Schultz K, Kramer AT. 2023. Variation in overall fitness due to seed source: projections for predictive provenancing. Restoration Ecology 31:e13717.

[CIT0123] Zeng X , Fischer GA. 2021. Using multiple seedlots in restoration planting enhances genetic diversity compared to natural regeneration in fragmented tropical forests. Forest Ecology and Management 482:118819.

[CIT0125] Zucchi MI , Sujii PS, Mori GM, Viana JPG, Grando C, Silvestre Ellida de Aguiar, Schwarcz KD, Macrini CM, Bajay MM, Araújo FL, et al. 2018. Genetic diversity of reintroduced tree populations in restoration plantations of the Brazilian Atlantic Forest. Restoration Ecology 26:694–701.

[CIT0124] Zuur AF , Ieno EN, Walker NJ, Saveliev AA, Smith GM. 2009. Mixed effects models and extensions in ecology with R (Vol. 574). NY: Springer.

