# Supplementary material for: Guiding seed movement: environmental heterogeneity drives genetic differentiation in Plathymenia reticulata, providing insights for restoration
Source: AoB Plants. 2024 May 29;16(3):plae032. doi: 10.1093/aobpla/plae032 (PMC11176975; doi:10.1093/aobpla/plae032)
Supplement: plae032_suppl_Supplementary_Material [file plae032_suppl_supplementary_material.pdf]

Guiding Seed Movement: Environmental Heterogeneity Drives Genetic Differentiation in *Plathymenia reticulata* Benth., Providing Insights for Restoration

Supplementary material

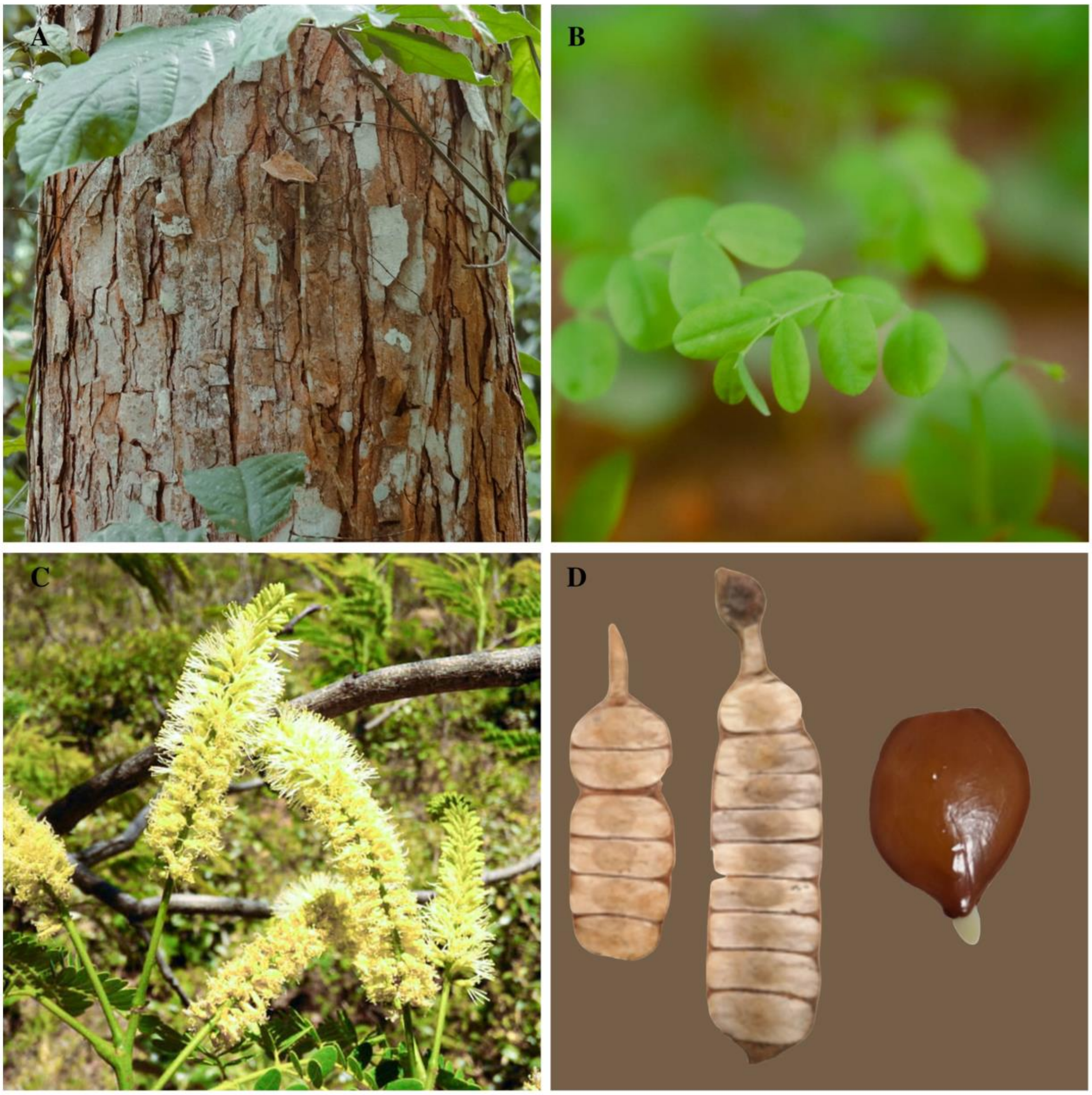

Fig S1. Different structures of *Plathymenia reticulata*. A: Trunk; B: Leaves; C: inflorescence; D: Fruits and seeds surrounded by membrane and seed with radicle protrusion. The fruits measure approximately 7.8–16 × 1.4–3.6 cm, the seeds 6–14×4–8 mm.

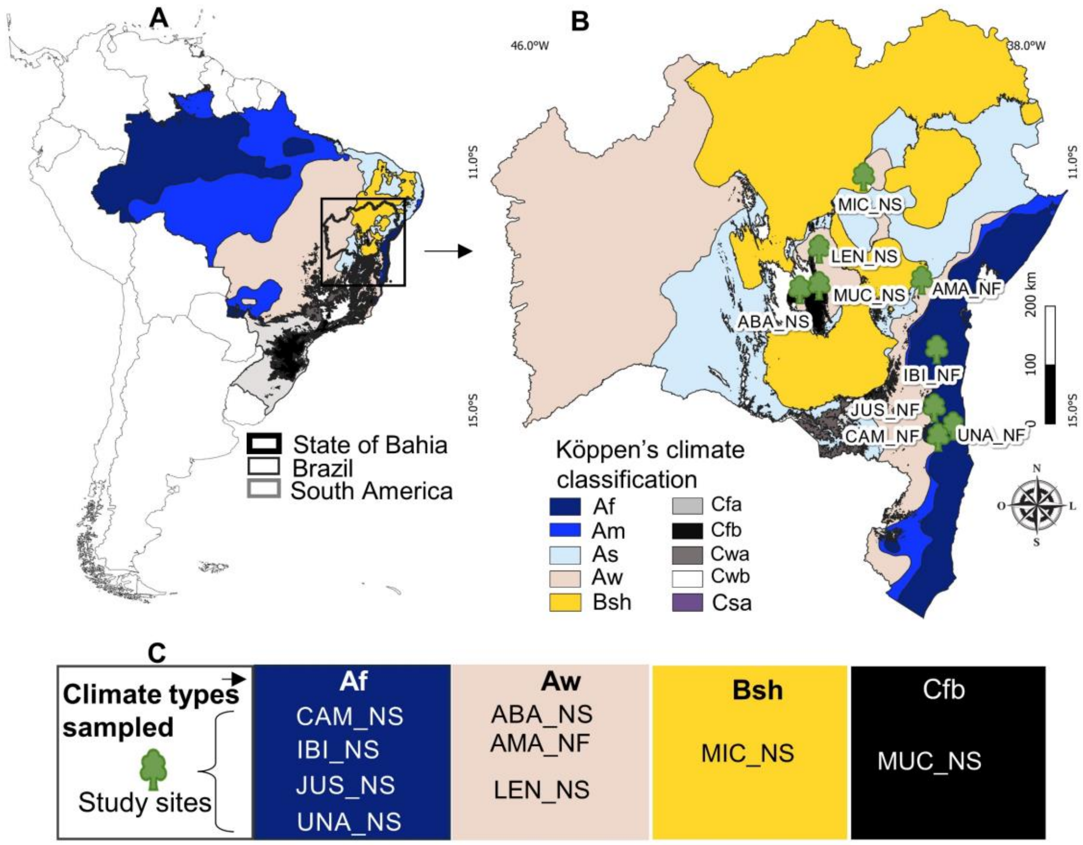

Fig S2. Map of South America, highlighting Brazil, Bahia and their climate types. (A) Koppen climate classification for Brazil; (B) geographic location of sampled populations of *Plathymenia reticulata* in climate types Af, Aw, Cfb and Bsh in Bahia, according to Koppen's classification for Brazil (Alvares et al., 2013); (C) sampling scheme with the climatic types studied Af (dark blue), Aw (beige), Cfb (black) and Bsh (yellow) with the populations that compose them. CAM_NF = Camacan, IBI_NF = Ibirapitanga, JUS_NF = Jussarí, UNA_NF = UNA, AMA_NF = Amargosa, ABA_NS = Abaíra, LEN_NS = Lençóis, MIG_NS = Miguel Calmon and MUC_NS = Mucugê. NF= Native Forest and NS= Native Savanna.

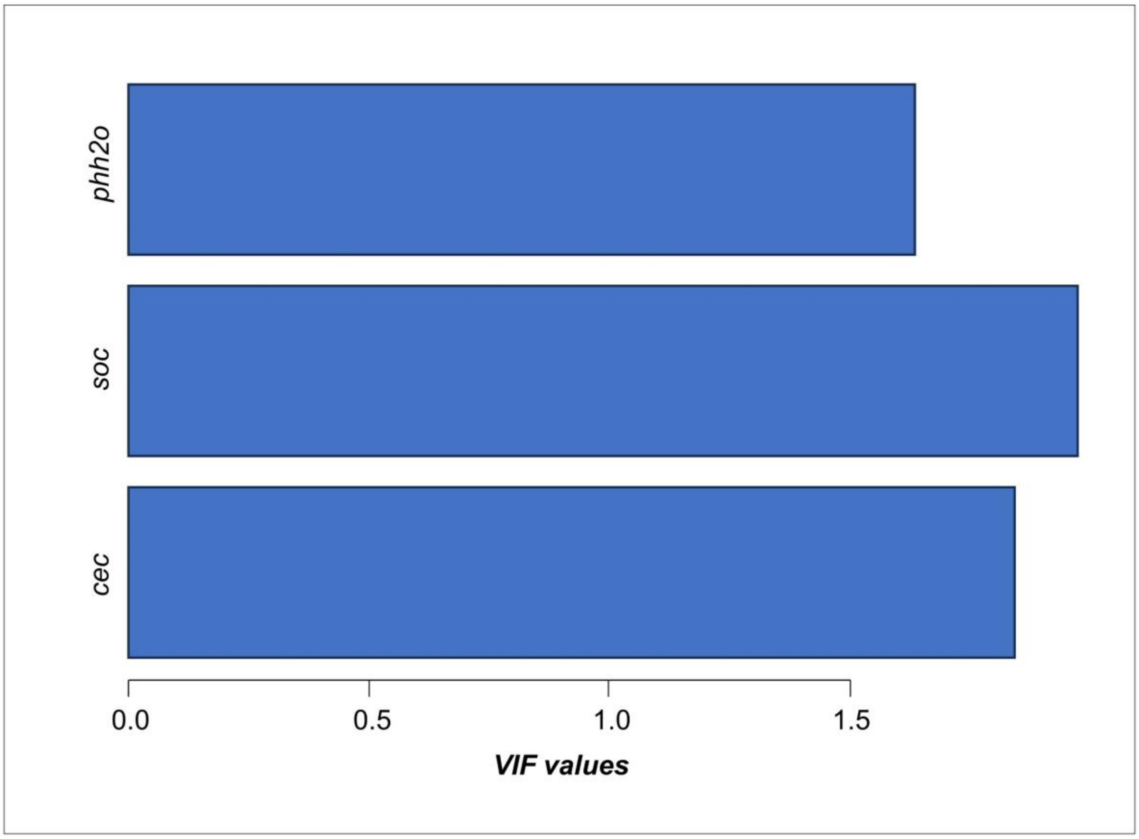

Fig S3. Correlation between edaphic variables. Values of variation inflation factor (*VIF*) for soil variables. *Cec*= soil cation exchange capacity; *phh2o*= soil pH; *soc*= soil organic carbon content. *Cec*, phh2o and soc present *VIF* values $\leq$ 5.

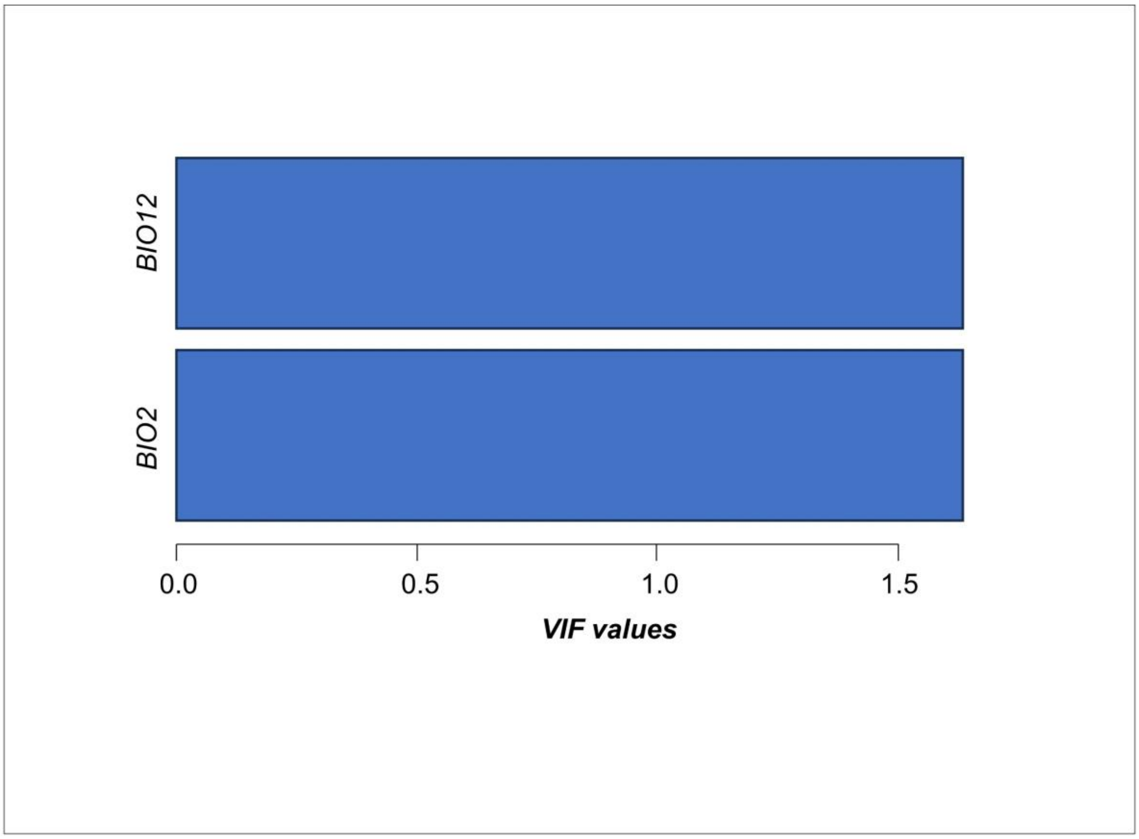

Fig S4. Correlation between bioclimatic variables Values of variation inflation factor (*VIF*) for clim variables. *BIO2* =Mean Diurnal Range; *BIO12*=Annual Precipitation. *BIO2* and *BIO12* present *VIF* values ≤ 5.

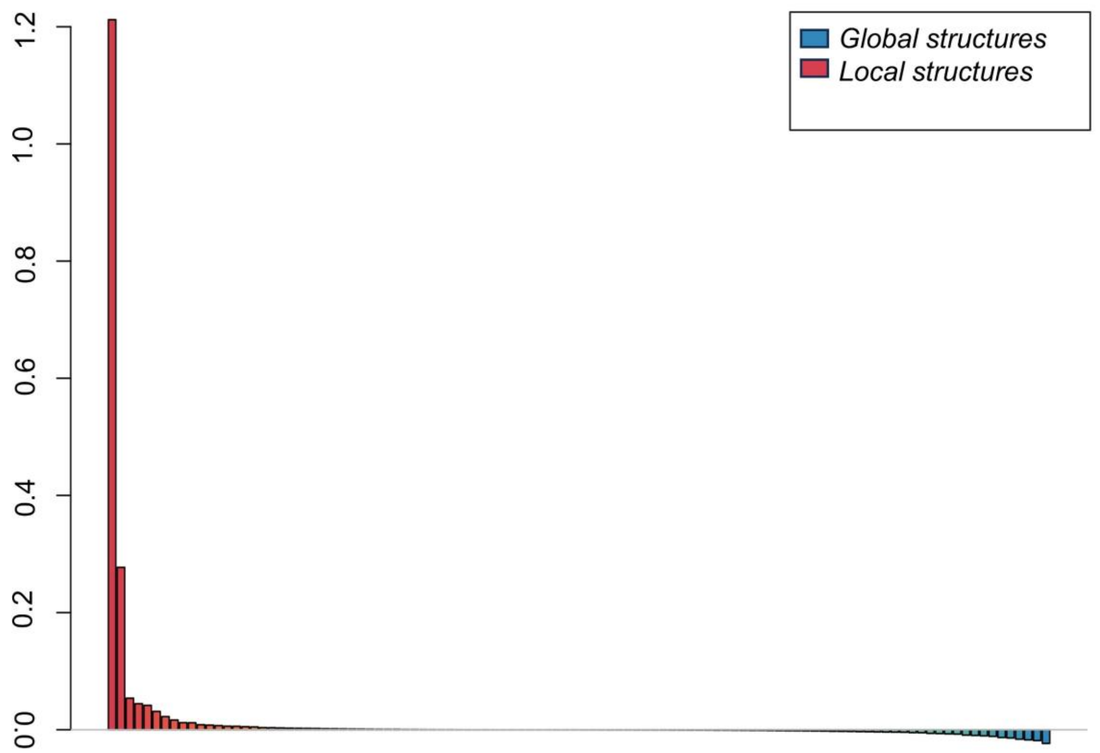

Fig S5. Genetic spatial principal component analysis (*sPCA*) for nine populations of *P. reticulata* in Bahia, Brazil, with the scores for each *sPCA* axis explaining local (blue) and global (red) genetic structure. As the first two axes of the *sPCA* presented the highest scores, they were used as a measure of the genetic structure in distance-based redundancy analysis (*dbRDA*).

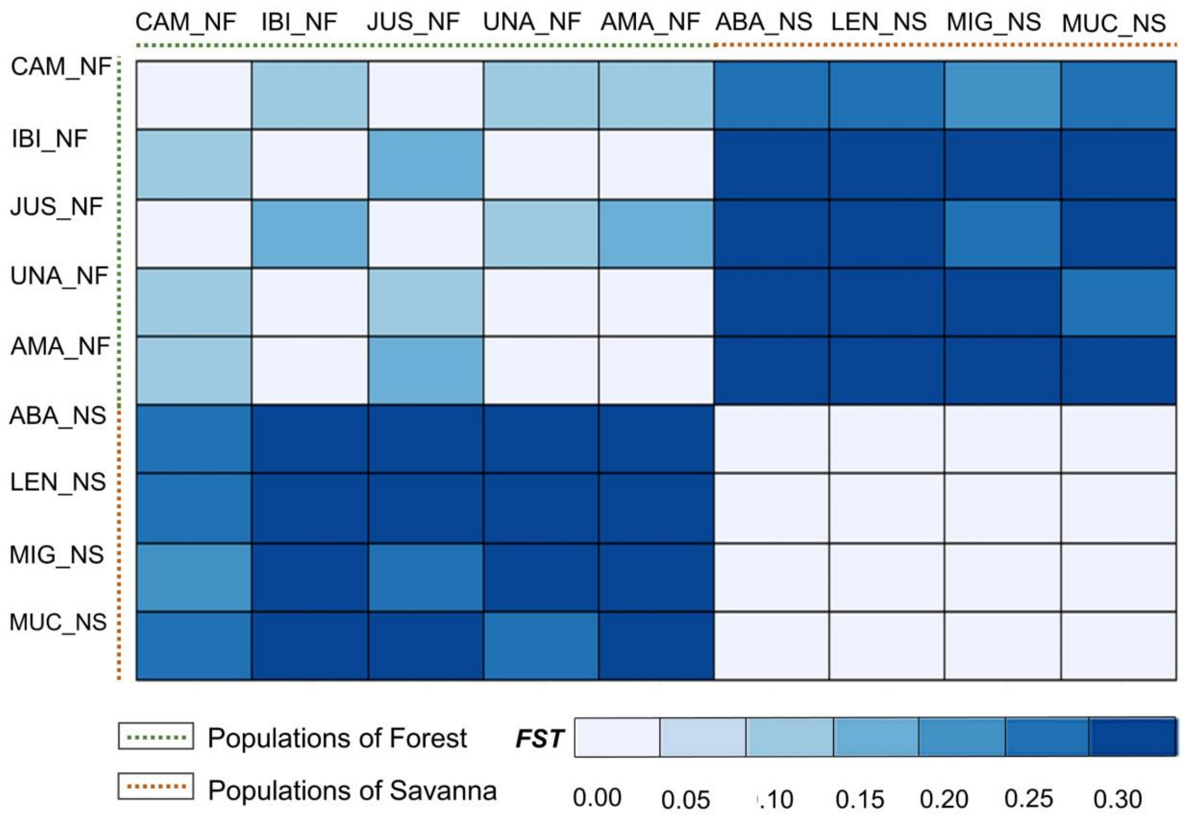

Fig S6. Pairwise F_{ST} estimated with nuclear microsatellite markers (nSSR) for nine populations of *P. reticulata* in Bahia, Brazil. Lighter blues show lower F_{ST} values, and darker tones show higher F_{ST} values. CAM_NF, IBI_NF, JUS_NF, UNA_NF and AMA_NF represent populations of Forest. ABA_NS, LEN_NS, MIG_NS and MUC_NS represent populations of Savanna.

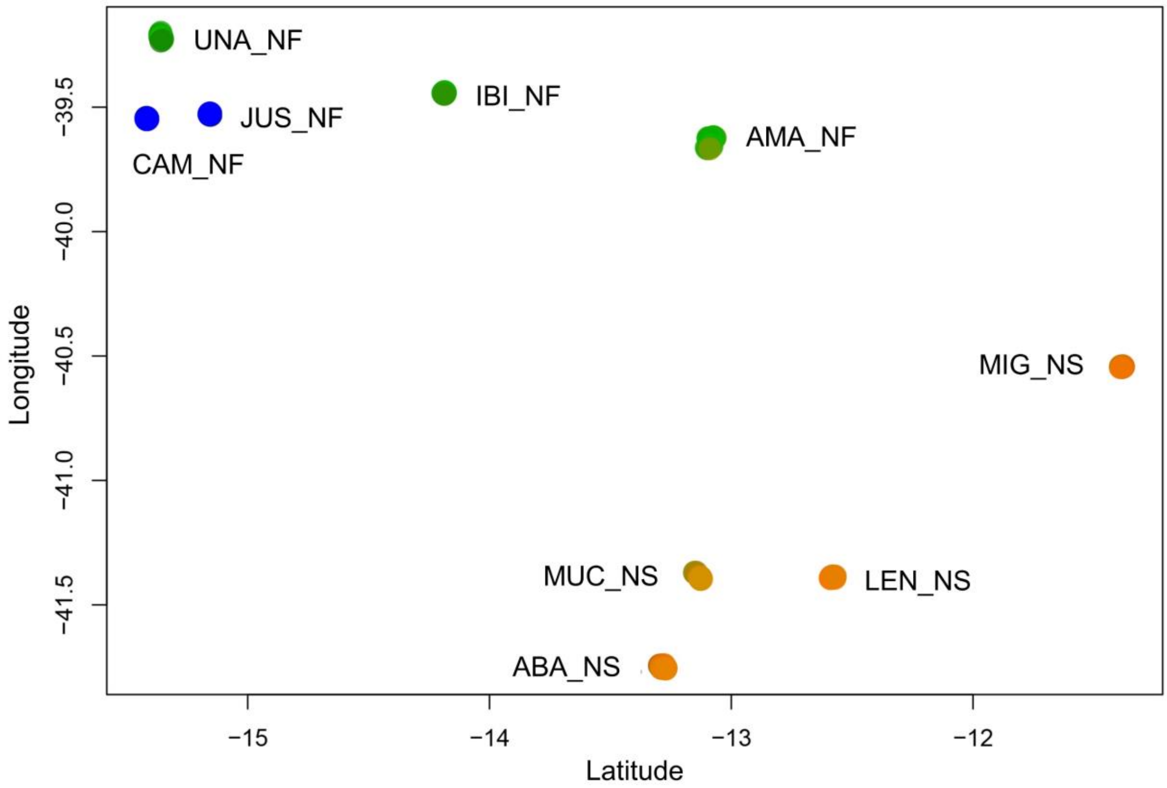

Fig S7. Spatial genetic structure with nuclear microsatellite markers (nSSR) for nine populations of *P. reticulata* in Bahia, Brazil, based on the first two scores of the genetic spatial principal component analysis (*sPCA*). Evidencing K=2, with a group composed of populations located in Savanna and another group formed by populations located in Forest (composed of two subgroups, highlighted in green and blue). CAM_NF = Camacan, IBI_NF = Ibirapitanga, JUS_NF = Jussarí, UNA_NF = UNA, AMA_NF = Amargosa, ABA_NS = Abaíra, LEN_NS = Lençóis, MIG_NS = Miguel Calmon and MUC_NS = Mucugê. NF= Native Forest and NS= Native Savanna.

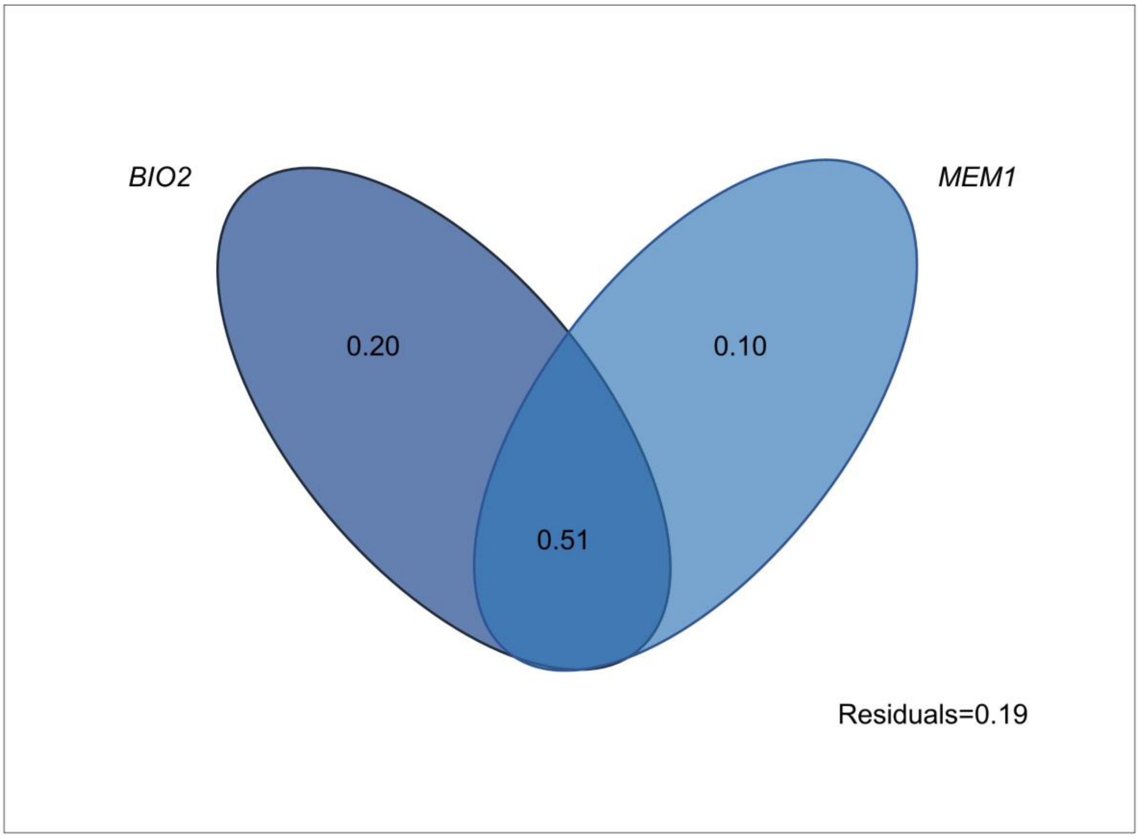

Fig S8. Venn diagram with the proportion of variance in genetic structure for nine populations of *P. reticulata*, explained by spatial variation (spatial predictor, *MEM1*) and *BIO2* = Average Diurnal Interval (maximum temperature - minimum temperature).

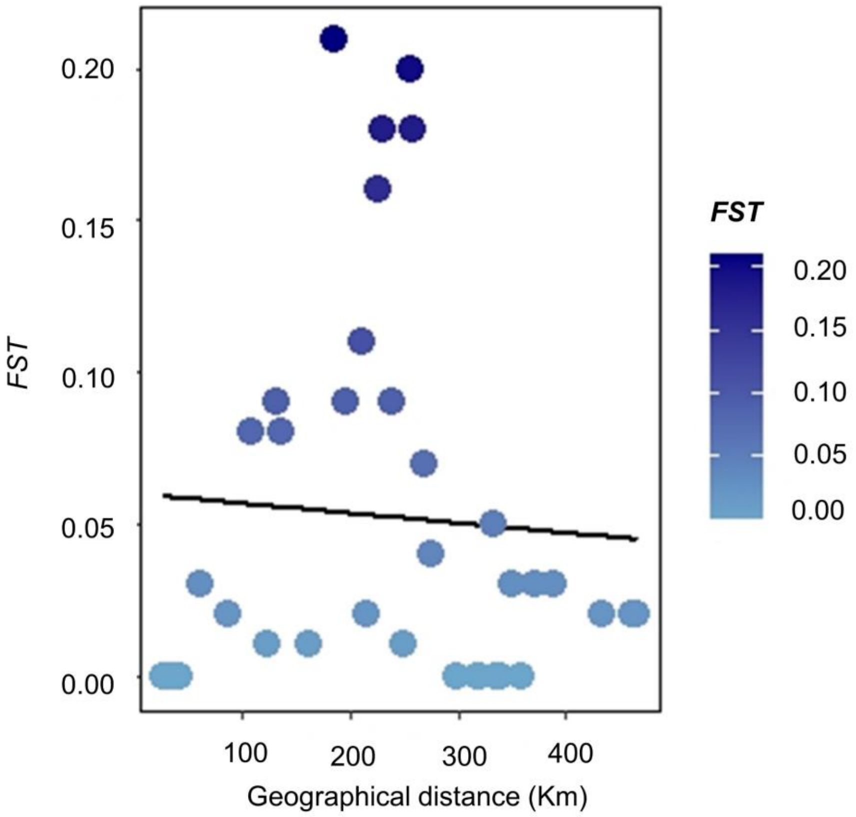

Fig S9. Simple Mantel test used to explore the relationship between genetic distance (F_{ST}) and geographic distance (Km) between pairs of nine populations of *P. reticulata* in Bahia, Brazil, by chloroplast microsatellite markers (cpSSR). (r = -0.06, P = 0.542).

Table S1. Analysis of molecular variance (AMOVA) for nine populations of *P. reticulata*, inserted in two phytophysiognomy (Forest and Savanna) and four climates (Af, Aw, Cfb and Bsh) in the state of Bahia, Brazil.

**Source of variation/ Phytophysiognomy**	**d.f.**	**Sum of squared deviation**	**Percentage of total variance**	**F-statistics**
Among phytophysiognomy	1	231.704	40.28	F_{CT} = 0.402*

Populations within phytophysiognomy	7	85.843	9.16	F_{SC} = 0.153*
Individuals within populations	473	543.606	50.56	F_{ST} = 0.494*
Total	481	861.154		
**Source of variation/Climatic typology**				
Among climate	3	167.656	12.10	F_{CT} = 0.121
Populations within climate	5	149.892	28.27	F_{SC} = 0.321*
Individuals within populations	473	543.606	59.63	F_{ST} = 0.404*
Total	481	861.154		

* Represent *F-statistics* which are significant (p- value < 0.05).

Table S2. Simple Mantel test for genetic, environment (edaphic and bioclimatic), and spatial distance for nine populations of *P. reticulata* in Bahia, Brazil.

**Test**	**Correlation**	***p***
Genetic × Edaphic	0.64	0.006*
Genetic × Bioclimatic	0.78	0.007*
Genetic × Spatial distance	0.67	0.01*

* p- value < 0.05

Table S3. Variables identified in distance-based redundancy analysis (*dbRDA*) as significantly associated with *P. reticulata* gene pool distribution in the populations evaluated in the study.

**Variables**	R^2 **adj**	***F*-value**	***p*-value**
MEM1	0.80	4.53	0.04
BIO2	0.71	20.37	0.004

MEM1 = spatial predictor selected in Moran's spatial eigenvector analysis; *BIO2* = Average Diurnal Interval (maximum temperature - min temperature).

Table S4. Proportion of the genetic structure of the nine *P. reticula* populations sampled in Bahia, Brazil, explained by the *RDA1* and *RDA2* axes and the relationship of *BIO2* and *MEM1* with the *RDA1* and *RDA2* axes, identified in distance-based redundancy analysis (*dbRDA*).

	Variance	F	Pr(>F)	Proportion Explained
RDA1	1.28038	146.581	0.033 *	0.83
RDA2	0.26215	30.012	0.327	0.17

	RDA1	*RDA2*
BIO2	0.9866	0.1632
MEM1	0.9378	-0.3472

*p-value < 0.05

Table S5. Haplotype frequency observed in *P. reticulata* according to populations, Forest and Savanna, and in climatic groups in Bahia, Brazil. CAM_NF = Camacan, IBI_NF = Ibirapitanga, JUS_NF = Jussarí, UNA_NF = UNA, AMA_NF = Amargosa, ABA_NS = Abaíra, LEN_NS = Lençóis, MIG_NS = Miguel Calmon and MUC_NS = Mucugê. NF= Native Forest and NS= Native Savanna.

Haplotype/Population	haplo-1	haplo-2	haplo-3	haplo-4
CAM_NF	0.00	1.00	0.00	0.00
IBI_NF	0.00	0.36	0.64	0.00
JUS_NF	0.00	1.00	0.00	0.00
UNA_NF	0.00	1.00	0.00	0.00
AMA_NF	0.00	0.04	0.96	0.00
ABA_NS	0.10	0.90	0.00	0.00

LEN_NS	0.04	0.65	0.00	0.31
MIG_NS	0.25	0.75	0.00	0.00
MUC_NS	0.00	1.00	0.00	0.00

Vegetation typology	haplo-1	haplo-2	haplo-3	haplo-4
Forest	0.0	0.7	0.3	0.0
Savanna	0.1	0.8	0.0	0.1

Climatic typology	haplo-1	haplo-2	haplo-3	haplo-4
Af	0.00	0.86	0.14	0.00
Aw	0.05	0.57	0.28	0.10
Bsh	0.25	0.75	0.00	0.00
Cfb	0.00	1.00	0.00	0.00

Table S6. Haplotypic diversity parameters for the *P. reticulata* populations studied based on three chloroplast simple sequence repeats (cpSSR): N = number of individuals, N_H = number of haplotypes, H_{Ne} = effective number of haplotypes, P_H = private haplotypes, H= Haplotypic richness and H_E= haplotypic diversity and D^2sh= distance between individuals. CAM_NF = Camacan, IBI_NF = Ibirapitanga, JUS_NF = Jussarí, UNA_NF = UNA, AMA_NF = Amargosa, ABA_NS = Abaíra, LEN_NS = Lençóis, MIG_NS = Miguel Calmon and MUC_NS = Mucugê. NF= Native Forest and NS= Native Savanna.

Population	N	N_H	H_{Ne}	P_H	H	H_E	D^2sh
CAM_NF	24	1	1	0	0	0	0.00
IBI_NF	22	2	1.86	0	1	0.48	0.16
JUS_NF	24	1	1	0	0	0	0.00
UNA_NF	27	1	1	0	0	0	0.00

AMA_NF	23	2	1.09	0	0.96	0.09	0.03
ABA_NS	30	2	1.22	0	0.99	0.19	0.06
LEN_NS	26	3	1.91	1	1.85	0.5	0.58
MIG_NS	28	2	1.6	0	1	0.39	0.13
MUC_NS	28	1	1	0	0	0	0.00
Population Mean	26	1.7	1.3	0.11	0.64	0.18	0.11

Table S7. Haplotypic diversity parameters for the *P. reticulata* populations in different Vegetation and Climatic typology studied based on three chloroplast simple sequence repeats (cpSSR): *N*= number of individuals, N_H = number of haplotypes, H_{Ne} = effective number of haplotypes, P_H = private haplotypes, *H*= Haplotypic richness, $H_{E=}$ haplotypic diversity and D^2sh= distance between individuals.

Vegetation typology	*N*	N_H	H_{Ne}	P_H	*H*	H_E	D^2sh
Forest	120	2	1.72	1	1	0.42	0.14
Savanna	112	3	1.42	2	2	0.30	0.22
Mean	116	2.50	1.57	1.50	1.50	0.36	0.18
**Climatic typology**	*N*	N_H	H_{Ne}	P_H	*H*	H_E	D^2sh
Af	97	2	1.33	0	0.99	0.25	0.08
Aw	79	4	2.41	1	2.81	0.59	0.50
Bsh	28	2	1.60	0	1.00	0.39	0.13
Cfb	28	1	1.00	0	0.00	0.00	0.00
Mean	58.00	2.25	1.59	0.25	1.20	0.31	0.18

Table S8. Normalized Pairwise F_{ST} with cpSSR for nine populations of *P. reticulata* in Bahia, Brazil. CAM_NF = Camacan, IBI_NF = Ibirapitanga, JUS_NF = Jussarí, UNA_NF = UNA, AMA_NF = Amargosa, ABA_NS = Abaíra, LEN_NS = Lençóis, MIG_NS = Miguel Calmon and MUC_NS = Mucugê. NF= Native Forest and NS= Native Savanna.

Populations	CAM_NF	IBI_NF	JUS_NF	UNA_NF	AMA_NF	ABA_NS	LEN_NS	MIG_NS	MUC_NS
CAM_NF	0.00								
IBI_NF	0.08	0.00							
JUS_NF	0.00	0.08	0.00						
UNA_NF	0.00	0.09	0.00	0.00					
AMA_NF	0.18	0.01	0.18	0.20	0.00				
ABA_NS	0.00	0.07	0.00	0.00	0.16	0.00			
LEN_NS	0.03	0.04	0.03	0.03	0.09	0.02	0.00		
MIG_NS	0.02	0.05	0.02	0.02	0.11	0.01	0.01	0.00	
MUC_NS	0.00	0.09	0.00	0.00	0.21	0.00	0.03	0.02	0.00
